# Spectroscopy-informed XANES–PXRD framework for multi-property prediction and structure inference

**DOI:** 10.1039/d6sc00651e

**Published:** 2026-05-20

**Authors:** Yang Wang, Siyuan Zhao, Man Luo, Hantao Zeng, Yi Feng, Daobin Liu, Yan Huang, Jun Jiang

**Affiliations:** a State Key Laboratory of Precision and Intelligent Chemistry, University of Science and Technology of China Hefei Anhui 230026 China ldbin@ustc.edu.cn hyan@ustc.edu.cn jiangj1@ustc.edu.cn

## Abstract

Accelerating functional material characterization and rational design faces a fundamental circular bottleneck in which all mainstream computational screening methods rely entirely on *a priori* crystallographic knowledge, information inherently unavailable for novel, uncharacterized samples. To bypass this bottleneck, we utilize spectroscopy as a predictive driver. While X-ray absorption near-edge structure (XANES) captures element-specific local states, powder X-ray diffraction (PXRD) resolves global long-range order. Here, we integrate XANES and PXRD into a unified spectroscopic representation to address the challenge of structure inference from spectral data. Trained on 34 929 inorganic compounds with over 100 000 simulated spectra, our framework jointly predicts key physical properties (*e.g.*, band gap, magnetism, density) while inferring oxidation states, coordination numbers, and crystal systems. Notably, a composition-aware partial-measurement strategy utilizing only transition-metal edges matches the accuracy of all-element models, significantly reducing experimental burden. Interpretability analyses reveal that transition-metal and non-transition-metal features cooperatively encode the correlations linking local electronic motifs to global symmetry. Crucially, the framework goes beyond property mapping to reconstruct charge-balanced formulas and retrieve structural templates from existing databases, enabling structure mining without prior structural knowledge. This approach achieves a top-1 accuracy exceeding 0.80 across binary to quinary systems, validated experimentally on eight representative samples including single-phase compounds and heterogeneous composites. These results establish spectroscopy as a quantitative, interpretable medium for decoding structure–property relationships, offering a practical pathway for spectroscopy-informed materials characterization and structure mining.

## Abstract 2

A unified AI framework integrating XANES and PXRD enables property prediction, local structure and crystal system inference, facilitating materials characterization without prior crystallographic knowledge.

## Graphical abstract

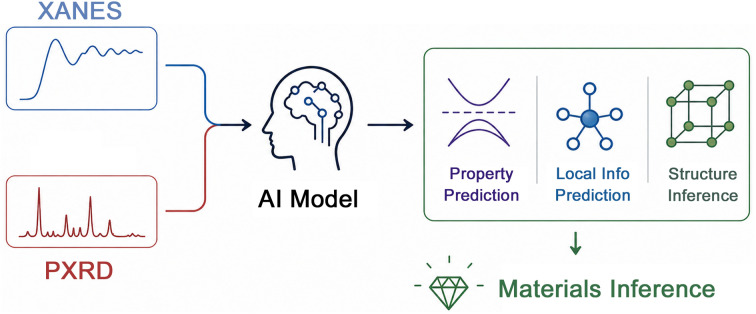

## Introduction

Accelerating functional material characterization and rational design is fundamental to tracing the origin of material properties and addressing critical challenges in energy and environmental applications.^1–4^ However, efficiently navigating the immense chemical space remains a formidable bottleneck. Traditional screening paradigms predominantly rely on structure-based approaches, utilizing first-principles calculations or machine learning models that require precise crystallographic information as input.^5–8^ This reliance creates a circular dependency in the exploration of novel materials: accurate property predictions necessitate *a priori* structural knowledge, yet such information is inherently unavailable for new, uncharacterized samples. Consequently, there is an urgent need for a high-throughput medium that can bypass this structural bottleneck. To break this deadlock, we propose to utilize spectroscopic signals not merely as diagnostic tools for post-synthesis characterization, but as predictive drivers for property prediction and structure inference. Spectroscopic data, such as X-ray absorption or diffraction patterns, serve as unique fingerprints that encode the intrinsic coupling between electronic states and atomic arrangements.^9–11^ By decoding these signals directly, we can establish a spectroscopy-driven structure inference workflow, transforming spectra into high-throughput search queries that bridge the gap between raw experimental signals and the physical understanding required for functional design.

Among various spectroscopic techniques, XANES spectroscopy serves as a crucial window into the intrinsic properties of materials owing to its element selectivity and high sensitivity to local electronic states.^12,13^ The systematic variations in edge energy and white-line intensity directly reflect the density of unoccupied states and the distribution of electronic states near the Fermi level, providing quantitative insights into band gap evolution and carrier characteristics.^14–16^ The pre-edge and near-edge features are highly sensitive to crystal-field splitting, exchange interactions, and spin-state transitions, thereby elucidating the formation mechanisms of magnetic ordering and spin polarization. Meanwhile, multiple scattering processes in the near-edge region respond to changes in bond lengths, bond angles, and metal–ligand interaction strength, mapping the stability of local configurations and the thermodynamic trends governed by the competition between enthalpy and entropy. In this way, XANES not only traces the evolution of valence states and coordination geometries but also encodes, within its spectral features, essential information about key physical properties such as band structure, magnetism, and stability.^17–19^ However, its information content remains primarily confined to the local scale and cannot independently resolve global structural attributes such as long-range lattice periodicity, space-group symmetry, phase separation, or superstructure modulation, thus precluding the reconstruction of a complete crystallographic picture from XANES alone.

To overcome the limitations of XANES in capturing long-range order and global symmetry, PXRD provides a crucial channel for accessing comprehensive crystallographic information. PXRD directly resolves the space group, lattice parameters, and phase composition of a material, quantifies long-range ordering and microstrain, and thereby establishes its symmetry constraints and structural classification.^20–24^ As such, XANES and PXRD are intrinsically complementary in their informational hierarchy.^25–28^ The former probes local electronic states and coordination geometries, while the latter anchors global crystallographic systems, lattice constants, and phase relationships. Joint analysis of these two modalities enables bidirectional mapping between spectra and both structure and properties within a unified spectroscopic representation space. This integration not only supports multitask prediction of key properties such as band structure, magnetic ordering, and thermodynamic stability but also allows structural inference under the combined constraints of global symmetry and local coordination. Together they lay the foundation for constructing a unified framework that links structure, electronic states, and material functionality.

Despite the highly complementary nature of XANES and PXRD, few machine learning studies have fully integrated these two modalities for joint property prediction and structure inference. Most existing efforts focus on single-spectroscopy inputs. For instance, a recent diffusion-model-based approach developed by Guo *et al.* enables *ab initio* structure solution directly from nanocrystalline powder diffraction patterns, yet this method relies exclusively on PXRD data and does not incorporate element-specific local electronic and coordination information from XANES.^29^ Other multimodal attempts are either limited to narrow material systems, lack the ability to simultaneously predict functional properties and infer multiscale structural descriptors, or require full-element spectroscopic measurements that impose heavy experimental burdens.

**Fig. 1:**
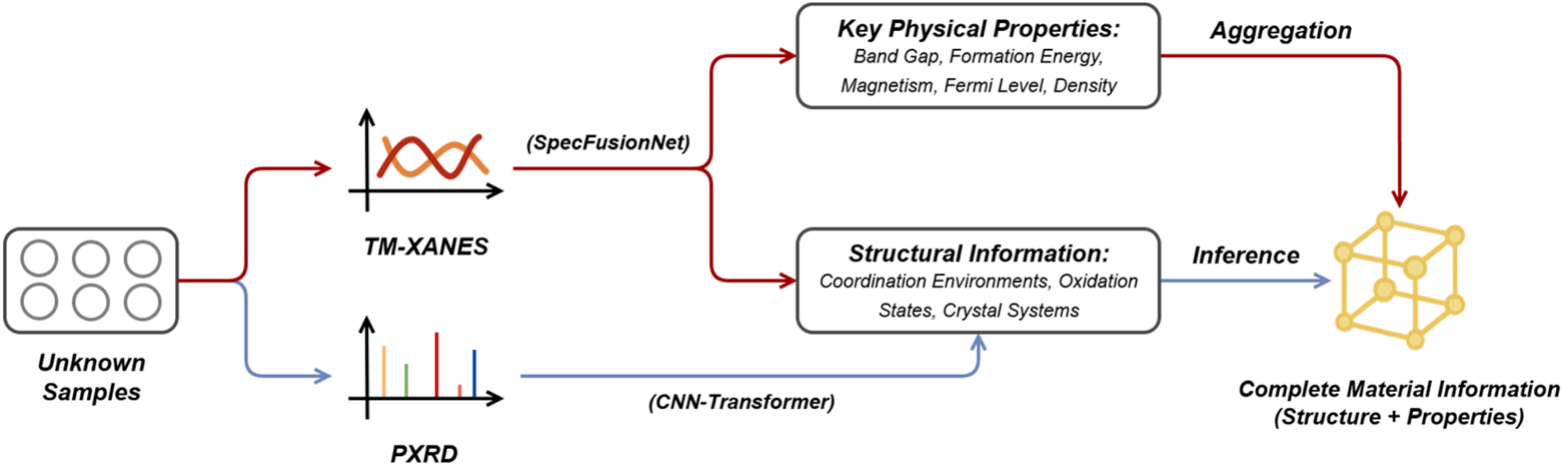
Integrated XANES–PXRD framework for multi property prediction and structure inference. Unknown samples are described by TM-XANES and PXRD spectra, which can be either simulated or experimentally measured. TM-XANES spectra are encoded by SpecFusionNet to predict physical and local structural information, while PXRD patterns are encoded by a CNN-transformer to classify crystal systems. The combined information enables structural-template retrieval and inference of complete material structures, providing a unified description of both structure and properties.

In this study, we propose an integrated framework that deeply fuses XANES and PXRD to quantitatively link spectral features with material properties within a unified representation space (Fig. 1). To resolve the circular dependency of structure-based screening, the framework synergistically combines the local electronic structure reflected by transition metal XANES with the global crystallographic symmetry and long-range order revealed by PXRD, thereby constructing a generalizable spectrum-crystal joint representation. Building on this, we develop a Spectral Fusion Network (SpecFusionNet) to predict key physical and structural information including band gap, magnetism, formation energy, Fermi level, density, coordination number, and oxidation state, and employ a CNN-Transformer architecture for high-accuracy crystal system identification. This multi-task architecture allows us to bypass *a priori* structural inputs, driving prediction directly from experimental fingerprints. These multi-scale structural and property predictions serve as the foundation for downstream structure inference.

Completing the workflow, the inferred structural priors enable constraint-based screening of existing databases to retrieve candidate structures, thereby facilitating structure mining in the absence of prior structural knowledge. Ablation and attribution analyses further delineate the critical information regions, quantitatively elucidating how local electronic motifs and global crystallographic symmetry act cooperatively to govern material functionality. Importantly, the framework exhibits excellent cross-domain generalization across large-scale theoretical datasets and diverse experimental systems, ranging from single-phase compounds to heterogeneous composites and a composition-aware partial-measurement strategy utilizing only transition-metal edges achieves comparable accuracy to all-element models, significantly reducing experimental burden. This demonstrates that even partial spectroscopic inputs can effectively disentangle dominant electronic features within complex mixed-phase environments, thereby encoding multiscale structural hierarchies. In doing so, this work establishes spectroscopy as a predictive, quantitative, and interpretable tool for data-driven materials characterization and structure mining, offering a general route for structural decoding and property inference without reliance on pre-solved structures.

## Results and discussion

### XANES-based physical properties prediction

**Fig. 2:**
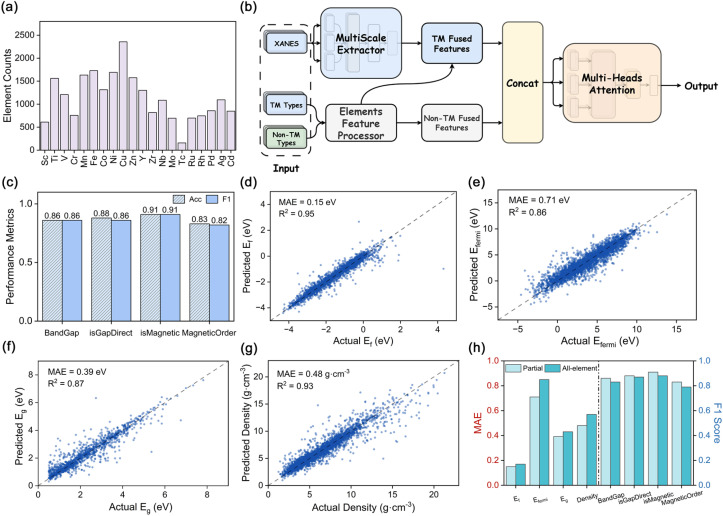
Prediction of physical properties using the XANES-based SpecFusionNet model. (a) Distribution of TM elements in the dataset, showing the frequency of occurrence for each species. (b) Architecture of the SpecFusionNet model. A multi-scale extractor captures fine-grained features from TM XANES spectra, while an element-feature processor encodes both TM and non-TM information. The fused representations are concatenated and passed through a multi-head attention module to generate property predictions. (c) Classification performance across four representative tasks: band gap presence (BandGap), direct *vs.* indirect gap (isGapDirect), magnetic presence (isMagnetic), and magnetic ordering (MagneticOrder), evaluated by accuracy and *F*1 score. (d–g) Parity plots of predicted *versus* actual values for four regression targets (*E*_f_, *E*_fermi_, *E*_g_, and density), with mean absolute error (MAE) and coefficients of determination (*R*^2^) annotated. (h) Comparison between models trained with all-element spectra and those using a partial-spectra input strategy, evaluated by MAE (left axis) and *F*1 score (right axis) across different prediction tasks.

To establish a comprehensive foundation for data-driven spectroscopic learning, we first constructed a large-scale theoretical dataset that systematically connects chemical composition, crystal structure, and electronic signatures. Specifically, we curated 34 929 inorganic compounds containing two to five elements from the Materials Project,^30,31^ and generated over 100 000 K-edge XANES spectra of transition-metal (TM) elements that span a wide chemical landscape encompassing oxides, sulfides, and mixed-anion systems (Fig. 2a and S1–S3). An additional dataset of all-element K-edge XANES spectra (including non-transition-metal constituents) was also prepared to construct a baseline model for performance comparison. Each XANES spectrum was preprocessed into 200 uniform intensity points within a 56 eV edge-centered window, and its fine structures were featurized *via* multi-scale convolution. For elemental featurization, we used fundamental atomic descriptors including atomic number, electronegativity, atomic radius, ionization energies, and element type. TM and non-TM elements are encoded separately.

To interpret these inputs, we developed the Spectral Fusion Network model, which serves as the XANES encoder within the framework (Fig. 2b). The model incorporates a multi-scale convolutional extractor that captures near-edge fine structures such as pre-edge and main-edge transitions at different resolutions (Fig. S4). In parallel, an element-feature encoder processes both TM and non-TM elemental information (Fig. S5). The spectral and compositional embeddings are then fused through a multi-head attention mechanism that adaptively weights each feature according to its relevance to the target property, enabling the model to learn physically interpretable, cross-scale correlations between local spectral fingerprints and physical properties.

To rigorously evaluate the predictive capacity of SpecFusionNet, we trained the model across a suite of classification and regression tasks encompassing diverse physical properties. The classification tasks encompassed the identification of band gap presence, direct *versus* indirect band gap, magnetism detection, and magnetic ordering, while the regression tasks targeted key quantitative descriptors including formation energy (*E*_f_), Fermi level (*E*_fermi_), band gap (*E*_g_), and density. These four classification labels and four regression targets span chemically diverse and moderately imbalanced distributions in band-gap character, magnetism, formation energy, Fermi level, band-gap magnitude, and density, thereby providing a stringent and representative test bed for evaluating SpecFusionNet (Fig. S6 and S7). Across all classification tasks, SpecFusionNet exhibits robust and transferable performance, with both accuracy and macro-*F*1 scores exceeding 0.82 (Fig. 2c and S8). For regression tasks, the model maintains low prediction errors, with mean absolute deviations below 0.71 eV for energetic quantities and 0.60 g cm^−3^ for density (Fig. 2d–g), underscoring its quantitative reliability. These results collectively confirm that transition-metal-centered XANES spectra encapsulate rich local electronic fingerprints that are sufficiently informative to reconstruct a broad spectrum of physical properties across chemically and structurally diverse materials, thereby establishing spectroscopy-driven learning as a powerful paradigm for universal materials representation and property inference.

We further benchmarked this configuration against an all-element baseline model that incorporated XANES spectra for every constituent element. The comparison revealed only marginal differences, with the mean absolute error (MAE) for band gap prediction increasing by merely 0.05 eV and classification *F*1 scores fluctuating by less than 6% (Fig. 2h). These results indicate that omitting non-transition-metal edges does not compromise predictive accuracy and may even enhance robustness by suppressing noise arising from low-information spectra. To further assess the generality of this strategy, we expanded the dataset to include K-edge XANES of alkaline-earth metals as well as L_2,3_-edge XANES of 5d transition metals and lanthanides. SpecFusionNet maintained consistently high predictive performance across these extended chemical systems (Fig. S9–S12), confirming that the composition-aware partial-measurement strategy provides a reliable and transferable foundation for quantitatively linking spectroscopic features with diverse physical properties.

### Interpretability of physical properties prediction

**Fig. 3:**
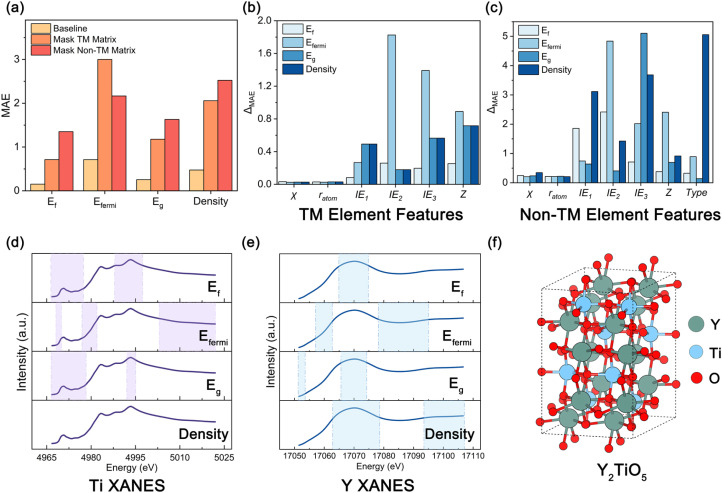
Interpretability and feature attribution in physical properties prediction. (a) Ablation analysis shows the effect of masking different element-fusion matrices on four regression targets. Bars compare the MAE of the baseline model with those obtained when the fusion matrices corresponding to TM or non-TM elements are masked. (b and c) Contribution of atomic descriptors for TM (b) and non-TM (c) elements to the four regression targets. Each bar represents the change in MAE (ΔMAE) relative to the baseline when the corresponding descriptor is removed. (d and e) Grad-CAM visualizations highlighting the importance of specific energy regions in the XANES spectra of Ti (d) and Y (e) in Y_2_TiO_5_ (mp-17559) for different regression tasks. The shaded areas denote spectral regions with normalized Grad-CAM values > 0.5, corresponding to the most influential features. (f) Ball and stick representation of the Y_2_TiO_5_ crystal structure, with Y, Ti, and O atoms shown in blue, green, and red, respectively.

To elucidate the role of localized spectroscopic information in physical properties prediction and to probe the internal attribution mechanisms of the SpecFusionNet, we conducted a series of interpretability analyses. Model performance was evaluated under two ablation settings in which either the TM-fusion or non-TM-fusion matrix was masked. In each case, the corresponding feature vectors were replaced with zeros while preserving tensor dimensions and parameter counts, thereby maintaining constant network complexity. Both ablations resulted in a marked decline in predictive accuracy (Fig. 3a and S13), revealing that transition-metal and non-transition-metal components act cooperatively to encode the key spectral–property correlations underpinning the predicted electronic and structural behaviors.

We further performed ablation analyses on the element-embedding features for both TM and non-TM components, focusing on key atomic descriptors including electronegativity (*χ*), atomic radius (*r*_atom_), ionization energies (IE_1_, IE_2_, IE_3_), atomic number (*Z*), and element type (type). Sequential removal of these descriptors elucidated their distinct physical contributions. Of these descriptors, *Z* exerted the most pervasive influence, as its elimination caused a pronounced increase in MAE across all prediction tasks, underscoring the fundamental role of nuclear charge and electronic configuration in determining physical properties. The type descriptor most strongly affected density prediction, consistent with its association with atomic packing motifs and bonding topology. Ionization energies were particularly important for band-structure-related quantities, while *χ* and *r*_atom_ contributed moderately yet significantly to properties governed by local coordination geometry and bond strength (Fig. 3b, c and S14). Collectively, these results quantitatively confirm that the learned elemental embeddings encode intrinsic chemical periodicity and its coupling with local spectral information, thereby enhancing the physical transparency and interpretability of the model.

To visualize how the model extracts property-specific spectral signatures, Grad-weighted class activation mapping (Grad-CAM) was applied to the final convolutional layers. For each spectrum, attention intensities were integrated over pre-edge, edge, and post-edge regions to identify the most influential energy intervals. In Y_2_TiO_5_ (mp-17559), both Ti and Y K-edges contributed substantially to the prediction of physical properties, with the Ti edge exhibiting enhanced pre-edge and main-edge activations that reflect the sensitivity of Ti 3d–O 2p hybridization to band-structure variations (Fig. 3d). The Y XANES spectrum shows a distinct activation pattern compared to Ti, and when predicting density, Grad-CAM highlights the Y K-edge almost exclusively, emphasizing the dominance of Y^3+^ coordination in determining lattice packing and overall cell volume (Fig. 3e). This trend is consistent with the crystal structure of Y_2_TiO_5_ (Fig. 3f). This task-dependent shift of spectral attention illustrates that the model adapts its learned representation to the physical origin of each property, with local orbital transitions governing band-gap estimation and heavy-atom coordination dominating density inference.

Interestingly, this element-specific dominance is not universal across all Y–Ti systems. A similar pattern was observed in Y_2_Ti_2_O_7_ and YTiO_3_, where both Y and Ti edges contributed prominently to the predicted density. Grad-CAM maps revealed comparable activation across the Ti pre-edge and Y main-edge regions, implying cooperative spectral influence between the two cations (Fig. S15 and S16). This behavior is consistent with the underlying crystal chemistry, as Ti primarily occupies octahedral sites whose connectivity modulates framework packing, while Y^3+^ coordination expands cell volume through its larger ionic radius.

Consequently, the model dynamically balances attention across multiple edges rather than privileging a single element, confirming that the learned XANES representation is chemically grounded and that element-resolved spectral features are quantitatively linked to macroscopic functionality.

### Multiscale structural descriptors from XANES and PXRD

**Fig. 4:**
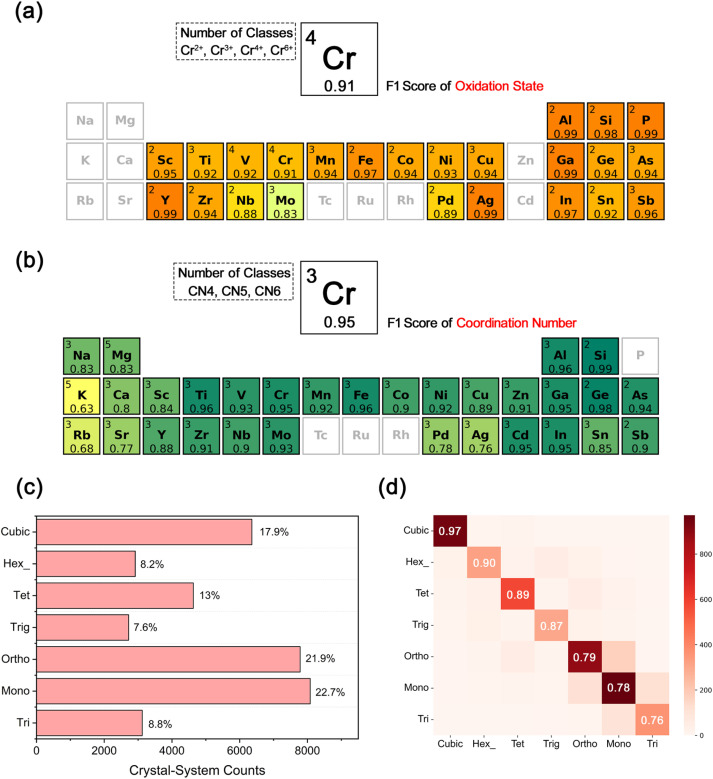
Prediction of local and global structural information from XANES and PXRD. (a) Element-wise oxidation-state classification based on XANES spectra. Each colored cell denotes an element with the number of oxidation states considered (upper left), the element symbol (center), and the corresponding macro-*F*1 score (bottom). Grey cells indicate elements with insufficient training data for reliable evaluation. (b) Element-wise coordination-number classification from XANES spectra. Each colored cell shows the number of coordination categories (*e.g.*, CN4–CN6), the element symbol, and the corresponding macro-*F*1 score. Grey cells mark elements without sufficient samples for quantitative evaluation. (c) Crystal system count distribution across the dataset, expressed as absolute counts of materials per crystal system. (d) Confusion matrix for crystal-system classification using PXRD-only inputs, yielding an overall accuracy of 0.81 across seven crystal systems. Darker diagonal cells correspond to higher correct-classification counts.

To extend our analysis from physical properties prediction to structural descriptors, we evaluated macro-*F*1 scores for oxidation-state and coordination-number classification using standardized XANES spectra for all transition-metal elements (Fig. S17). Most transition metals exhibit macro-*F*1 scores above 0.90 for both tasks, reflecting the pronounced sensitivity of near-edge features to variations in d-orbital occupancy and ligand-field geometry. The most discriminative features for oxidation-state prediction occur near the absorption edge, where the energy shift correlates with effective nuclear charge and covalency, whereas coordination-number recognition depends primarily on pre-edge and near-edge oscillations associated with crystal-field splitting. For main-group cations which lack d states, small but systematic edge shifts still enable reliable identification of coordination environments, particularly in distorted or non-octahedral sites (Fig. 4a and b). These results demonstrate that the approach generalizes beyond transition metals, revealing that near-edge spectral signatures carry chemically transferable descriptors of local structure across diverse chemistries.

Whereas XANES offers element-specific sensitivity to local coordination environments, PXRD encodes the long-range crystallographic symmetry and lattice periodicity that cannot be resolved from near-edge spectra alone. To assess how reliably such global symmetry information can be extracted under realistic measurement conditions, we first convolved the simulated PXRD patterns with a Voigt function to emulate instrumental peak broadening, then added controlled Gaussian white noise to mimic counting statistics and background fluctuations (Fig. S18). The resulting dataset spans seven representative crystal systems (Fig. 4c) and was used to train a CNN-Transformer classifier based solely on PXRD inputs (Fig. S19). Despite the presence of noise, the model attains an overall crystal-system classification accuracy of 0.81 (Fig. 4d). High-symmetry systems such as cubic and hexagonal are predicted with accuracies above 0.90, reflecting their relatively simple and well-separated diffraction signatures. By contrast, orthorhombic, monoclinic and triclinic systems reach more modest accuracies of 0.70–0.78, as their peak-rich patterns exhibit substantial overlap and subtle angular splittings (Fig. S20). The reduced performance for these low-symmetry systems arises from the intrinsic similarity of their diffraction profiles, in which weak symmetry-diagnostic reflections and minor intensity modulations are easily masked by the imposed noise, rendering symmetry-dependent distinctions challenging even in this idealized learning scenario.

To disentangle the roles of local and global spectroscopic information, we benchmarked models trained on XANES-only, PXRD-only, and fused XANES–PXRD inputs. PXRD delivers the highest accuracy (0.81), whereas fusion reaches 0.66 and XANES alone yields 0.48 (Fig. S21). The inferior accuracy of the fused model stems from the exclusive dependence of crystal-system classification on long-range symmetry information, which is uniquely encoded in PXRD patterns; the incorporation of XANES input introduces extraneous local structural descriptors that distort the identification of symmetry-diagnostic diffraction features. This hierarchy highlights the intrinsic complementarity of the two spectroscopic modalities: PXRD encodes lattice periodicity and global long-range symmetry, whereas XANES probes element-specific local coordination environments and oxidation-state variations. This performance distinction justifies our framework's design, which assigns crystal-system classification explicitly to the PXRD encoder to maximize accuracy. By integrating these complementary predictions in the downstream workflow, the framework establishes spectroscopy as a multiscale structural probe and provides a robust foundation for the subsequent structure-inference module.

### Structure inference and framework validation

Building on the multi-scale structural descriptors extracted from XANES and PXRD, the structure-inference module of our framework is designed as a conditional data-mining and structural-template retrieval tool to match the most plausible crystal structures under spectroscopic constraints.

**Fig. 5:**
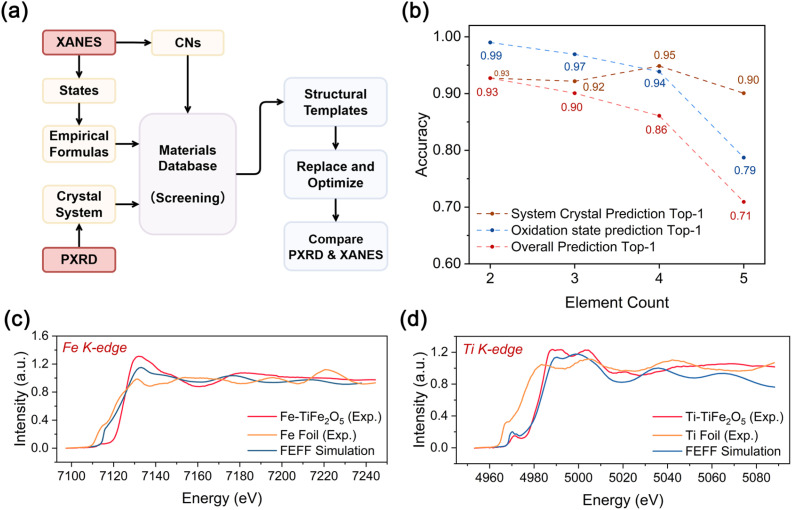
Structure inference and experimental validation of the integrated framework. (a) Schematic of the structure-inference stage, integrating predicted oxidation states, coordination numbers, and crystal systems to generate charge-balanced candidates. (b) Accuracy of empirical-formula prediction, crystal-system classification, and their combined top-1 performance as a function of element count (two to five). (c) and (d) Comparison of experimental, reference foil, and FEFF-simulated XANES spectra for TiFe_2_O_5_ at the Fe K-edge (c) and Ti K-edge (d), respectively. These spectra illustrate the cross-modal consistency between theoretical predictions and experimental results; all experimental spectra were background-subtracted and normalized for direct comparison.

As shown in Fig. 5a, the module takes three core predicted descriptors as input constraints: oxidation states and coordination numbers from the XANES encoder, and crystal system classification from the PXRD encoder, which together define the chemically feasible compositional and symmetry space for candidate screening. Within this constrained space, we first enumerate charge-balanced stoichiometries following integer-valence rules. We then retrieve representative crystallographic prototypes matching the predicted composition type and symmetry constraints from the full Materials Project inorganic crystal structure database (the module is also compatible with other mainstream crystallographic databases such as ICSD). Subsequently, elemental substitutions guided by coordination environment similarity and ionic radius compatibility are performed on the retrieved templates to generate chemically plausible candidate structures. Finally, all candidates are geometrically relaxed, and ranked by the consistency between their simulated spectra and the experimental input spectra, to identify the atomic configuration that best matches the experimentally inferred electronic and crystallographic signatures. Full details of the retrieval rules and workflow are provided in Sections S2.2 and S2.3 of the SI.

To evaluate the reliability of the inference module, we benchmarked its performance using theoretical spectra derived from first-principles calculations on the Materials Project dataset, with all evaluations conducted on its held-out theoretical subset. Across binary to quinary systems, the combined top-1 accuracy for simultaneous empirical-formula and crystal-system prediction exceeded 0.80 (Fig. 5b), indicating that the spectroscopic constraints are sufficient to recover both composition and symmetry with high fidelity. A representative example, Ca_2_MnAlO_5_, illustrates the entire reconstruction workflow. Under charge-balance and coordination constraints, two chemically plausible formulas, CaMnAlO_4_ and Ca_2_MnAlO_5_, were first enumerated (Fig. S22). For each candidate, representative structural models were generated and their PXRD patterns and XANES spectra were simulated. Direct comparison with the reference PXRD and XANES spectra unambiguously selected Ca_2_MnAlO_5_ as the correct solution (Fig. S23 and S24). Additional evaluations on three compounds excluded from the training set confirmed comparable reconstruction accuracy, demonstrating that the framework does not overfit to the training distribution and can be transferred to previously unseen chemical systems (Fig. S25). These results show that the inference module can reliably translate spectral information into chemically and structurally consistent atomic configurations.

The structure-inference module was further validated using seven experimentally synthesized compounds. Their XANES spectra were processed using identical normalization and interpolation protocols as those applied to theoretical data to ensure consistent spectral treatment across modalities (Fig. 5c, d and S26–S31). Across all samples, the experimental spectra closely reproduce the simulated XANES profiles in terms of edge positions, peak intensities, and fine-structure oscillations, demonstrating that the simulation pipeline faithfully captures the underlying electronic and coordination environments. Complementary PXRD measurements for the same samples reveal similarly high agreement between predicted and experimental diffraction patterns, further corroborating the structural assignments (Fig. S32). These results confirm that the integrated spectroscopic-inference framework generalizes from simulated to laboratory data with minimal loss of fidelity, enabling robust structure reconstruction under realistic experimental conditions.

To examine performance beyond structural concordance, we additionally evaluated property-level fidelity across the seven single-phase experimental validation samples. Band gaps and magnetic responses were measured by UV-vis diffuse reflectance and magnetic hysteresis loops, respectively (Fig. S33–S40), while other quantities were compared with theoretical references. The sample-wise prediction performance, including the consistency of structural classification tasks and the absolute error of quantitative property predictions, is summarized in Table 1. For structural classification tasks, the framework achieves 100% accuracy in crystal system prediction across all seven samples, with 6 out of 7 samples showing fully consistent oxidation state and coordination number predictions compared with experimental crystallographic results. For quantitative property prediction, the absolute errors are naturally larger than those on theoretical benchmarks due to domain shifts between simulated and experimental spectra. Specifically, band gap absolute errors range from 0.39 to 1.27 eV, density errors from 0.16 to 1.47 g cm^−3^, and formation energy errors remain below 0.24 eV for all samples. Despite the increased numerical errors, the model correctly identifies the trend of band gaps across the seven compounds and consistently distinguishes insulating from metallic behavior. Moreover, the formation energy predictions maintain a remarkably low absolute error (<0.24 eV) for all experimental samples, indicating that the learned spectral–energy mapping is robust against experimental noise. The full raw predicted and reference values for all properties are provided in Table S1 of the SI. A direct comparison between experimental and simulated XANES spectra shows that larger prediction errors correlate with greater spectral deviations, underscoring the sensitivity of the framework to experimental spectral fidelity.

**Table 1:** Prediction performance on seven experimental compounds^a^^,^^b^

Compound	*E*_f_	*E*_fermi_	*E*_g_	Density	Valence & CN	Crystal system
TiFe_2_O_5_	0.08	1.06	0.72	0.53	✓	✓
MnS	0.03	1.30	1.27	0.16	✓	✓
ZnFe_2_O_4_	0.10	0.65	0.64	0.97	✓	✓
CoFe_2_O_4_	0.02	1.26	0.50	1.37	✓	✓
V_2_O_5_	0.23	1.06	0.52	0.45	✓	✓
Co_2_O_3_	0.21	1.04	0.39	1.47	✓	✓
Co_3_O_4_	0.24	0.72	0.42	1.21	✗	✓

a Values shown as the absolute error between predicted and experimental/reference values.

b Units: formation energy (*E*_f_), Fermi level (*E*_fermi_), and band gap (*E*_g_) in eV; density in g cm^−3^. ✓ = predicted structural descriptors fully consistent with experimental crystallographic results; ✗ = partial inconsistency.

The only sample with partial inconsistency in oxidation state and coordination number assignments is Co_3_O_4_, a spinel-structured compound with two non-equivalent Co sites: tetrahedrally coordinated Co^2+^ and octahedrally coordinated Co^3+^. Its Co K-edge XANES spectrum is an inherent superposition of signals from these two mixed-valence, distinct coordination environments, which increases the difficulty of resolving two separate sets of local structural descriptors from a single, convoluted spectrum. This result further validates the high sensitivity of our model to element-specific local coordination and electronic states, and we have noted this specific challenge for multi-site mixed-valence systems as a key direction for future method optimization.

**Fig. 6:**
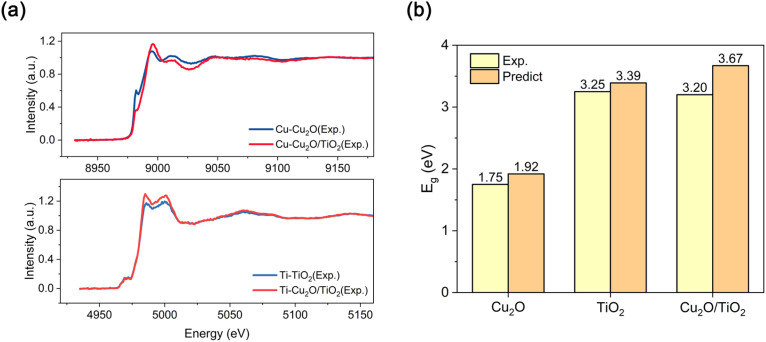
Experimental validation on a Cu_2_O/TiO_2_ heterogeneous composite. (a) Normalized experimental XANES spectra at the Cu K-edge (blue) and Ti K-edge (red) of the Cu_2_O/TiO_2_ composite. The Ti K-edge spectrum is dominated by features characteristic of TiO_2_, while the Cu K-edge shows contributions from Cu_2_O. (b) Comparison of the band gap predicted by the framework with the experimental value derived from UV-vis diffuse reflectance spectroscopy (Tauc plot method).

To further validate the generalization capability of the framework in heterogeneous systems, we investigated a Cu_2_O/TiO_2_ composite photocatalyst. While the material was fully characterized experimentally (PXRD and UV-vis spectra are shown in Fig. S41–S44), we utilized solely the experimental XANES spectra (Fig. 6a) as input to probe the electronic structure. The model successfully identified the electronic dominance of the wide-bandgap TiO_2_ component within the composite. As shown in Fig. 6b, the predicted band gap (3.67 eV) exhibits excellent agreement with the experimental trend derived from UV-vis measurements, proving that the model can extract critical electronic features even from the convoluted spectral signals of mixtures. This successful reconstruction underscores the framework's ability to bridge the gap between spectral diagnostics and physical inference, providing a robust tool for analyzing realistic, multi-component materials.

## Conclusions

This work establishes a unified XANES–PXRD framework that quantitatively links multi-scale spectroscopic features to both material properties and atomic structures. By integrating element-specific local electronic information from XANES with the global crystallographic symmetry captured by PXRD, the framework constructs a coherent representation that bridges the electronic and crystallographic domains. Within this representation, the model predicts diverse physical properties and structural descriptors, demonstrating the capability to disentangle dominant electronic signatures in heterogeneous systems, while a structure-inference module reconstructs charge-balanced formulas and retrieves structural templates under spectroscopic constraints. The framework achieves robust performance across theoretical and experimental domains, demonstrating accurate reconstruction of both electronic properties and crystal structures. While the current model exhibits high fidelity for broad material classes, we acknowledge challenges in differentiating complex low-symmetry systems or heavily disordered phases, highlighting areas for future algorithmic refinement.

Attribution and ablation analyses reveal that transition-metal and non-transition-metal features cooperatively encode the correlations that couple local electronic motifs with long-range symmetry, highlighting the physical interpretability of the model. These results suggest that spectroscopy can serve as a predictive, quantitative, and interpretable medium for decoding structure–property relationships, offering a practical framework for data-driven candidate screening and structure inference. Beyond the present results, the unified spectroscopic representation can be further extended to model task-specific properties across diverse materials domains. Crucially, by enabling the rapid identification of functional phases within complex or uncharacterized streams—such as industrial waste or low-value byproducts—this framework offers a tangible pathway for materials valorization, creating new opportunities for design from a circular-economy perspective.

## Methods

### Spectral preprocessing

To ensure consistent data quality, each XANES spectrum was interpolated within a 56 eV window centered at the absorption edge, yielding 200 uniformly spaced intensity points. Spectra were normalized to unit height and screened to remove artifacts or non-physical baselines. For PXRD, theoretical diffraction patterns were calculated over 10–90° (2*θ*) using relaxed crystal structures. The patterns were first broadened *via* convolution with a Voigt function (combining Gaussian and Lorentzian components) to simulate instrumental broadening, then resampled to 800 points. Subsequently, controlled Gaussian white noise was added to mimic experimental counting statistics and background fluctuations. This preprocessing procedure ensures that both XANES and PXRD datasets are standardized and noise-aware before model training.

### Model architecture

The integrated framework comprises two complementary encoders. The XANES encoder (SpecFusionNet) employs multiscale convolutional branches (kernel sizes = 3, 5, 7) to capture pre-edge, main-edge, and post-edge features at different resolutions. Elemental information, including atomic number, electronegativity, atomic radius, ionization energies, and categorical element type, is transformed through embedding layers and fused with spectral features *via* a multi-head attention module. This design allows both transition-metal and non-transition-metal elements to contribute chemical context, even when only partial XANES measurements are available. A masking mechanism enables compounds containing variable numbers of elements to be processed without introducing artifacts. The PXRD encoder adopts a CNN-Transformer architecture to capture both local peak correlations and long-range symmetry information. The two encoders jointly generate hierarchical representations that connect local electronic environments with global crystallographic order. Detailed layer configurations and hyperparameters are provided in the SI.

### Experimental details

PXRD patterns were recorded on a Rigaku SmartLab multifunctional rotating-anode diffractometer using Cu Kα radiation (*λ* = 1.54184 Å) operated at 45 kV and 200 mA. Data were collected in a continuous mode over a 2*θ* range of 10° to 90°, with a step size corresponding to a scanning speed of 10° min^−1^. X-ray absorption near-edge structure (XANES) spectroscopy was performed on a laboratory-based spectrometer (RapidXAFS 2 M, Anhui Absorption Spectroscopy Analysis Instrument Co., Ltd) equipped with a Mo target source operated at 20 kV and 20 mA. Energy selection was achieved using a spherically bent crystal analyzer (SBCA, 500 mm radius of curvature) chosen specifically for each absorption edge. Room-temperature magnetic hysteresis loops were measured using a Magnetic Property Measurement System (MPMS3, Quantum Design, USA). The magnetic field was swept from +50 000 Oe to −50 000 Oe and back to complete the loop. Solid-state UV-vis-NIR spectra were collected with a Shimadzu SOLID3700 spectrophotometer equipped with an integrating sphere for diffuse reflectance measurements over the 200–800 nm wavelength range. The diffuse reflectance data (*R*) were converted to the Kubelka–Munk function *F*(*R*) (1 − *R*)^2^/(2*R*). The Tauc plot was then obtained by plotting [*F*(*R*)*hν*]^2^*versus hν*, from which the band gap was determined by extrapolating the linear portion of the curve to the energy axis.

## Author contributions

J. J., Y. H., and D. L. conceived and supervised the project. Y. W. performed all data computation, model training, and framework design. S. Z. conducted the experimental measurements. Y. W., J. J., Y. H., and D. L. led the preparation of the manuscript with input from all other authors.

## Conflicts of interest

The authors declare no conflicts of interest.

## Supplementary Material

SC-OLF-D6SC00651E-s001

## Data Availability

The code and data underlying the machine learning part of this paper are available in our public repository (https://github.com/WangyLab/XANES-XRD_WorkFlow). Supplementary information (SI): additional methodological details (model training, evaluation protocol, and interpretability analyses); structure inference details (oxidation state determination, crystal system classification, template retrieval, DFT optimization, and spectral validation); computational details for XANES calculations and DFT structural optimization; Fig. S1–S44 and Table S1. See DOI: https://doi.org/10.1039/d6sc00651e.
