# Supplementary material for: Spectroscopy-informed XANES–PXRD framework for multi-property prediction and structure inference
Source: Chem Sci. 2026 May 20;17(26):13019–29. doi: 10.1039/d6sc00651e (PMC13202370; doi:10.1039/d6sc00651e)
Supplement: SC-017-D6SC00651E-s001 [file SC-017-D6SC00651E-s001.pdf]

Supplementary Information (SI) for Chemical Science.
This journal is © The Royal Society of Chemistry 2026

Supporting Information

Spectroscopy-Informed XANES–PXRD Framework for Multi-Property Prediction and Structure Inference

Yang Wang[1,#], Siyuan Zhao[1,#], Man Luo[1], Hantao Zeng[1], Yi Feng[1], Daobin Liu[1]*, Yan Huang[1]*, Jun Jiang[1]*

[1]State Key Laboratory of Precision and Intelligent Chemistry, University of Science and Technology of China, Hefei, Anhui 230026, China

[#]Y. W. and S. Z. contributed equally to this work.

*Correspondence: ldbin@ustc.edu.cn; hyan@ustc.edu.cn; jiangj1@ustc.edu.cn

This PDF file includes:

S1. Additional Methodological Details
- S1.1 Model Training and Evaluation Protocol
- S1.2 Ablation and Interpretability Analyses

S2. Structure Inference Details
- S2.1 Oxidation State Determination and Stoichiometric Formula Generation
- S2.2 Crystal System Classification and Template Retrieval
- S2.3 DFT Optimization and Spectral Validation
- S2.4 Handling Systems with Limited Templates

S3. Computational Details
- S3.1 XANES Calculations
- S3.2 DFT Structural Optimization

Figures S1 to S44 and Table S1

S1. Additional Methodological Details

S1.1 Model Training and Evaluation Protocol

All models were trained using a supervised learning strategy on datasets partitioned into 70% training, 15% validation, and 15% test subsets. The Adam optimizer with an initial learning rate of 10^{-3} was adopted, and L1 loss was used to measure prediction error, defined as the mean absolute difference between the predicted and reference values.

A masking mechanism ensured that zero-padded inputs (arising from variable element counts or spectra lengths) did not contribute to gradient updates. Training proceeded for up to 300 epochs with early stopping when validation loss plateaued for 20 epochs.

Each model's performance was quantified by MAE for regression tasks and by accuracy/F1 score for classification tasks. To evaluate robustness, five independent runs with different random seeds were performed; the average and standard deviation were reported.

S1.2 Ablation and Interpretability Analyses

To assess the role of individual components, ablation experiments were performed by selectively disabling modules in SpecFusionNet, including (i) the TM-XANES fusion block, (ii) the non-TM embedding branch, and (iii) atomic descriptor subsets (χ, r_{atom}, IE_1–IE_3, Z, Type). Removal of each component was compensated by zero-masking to maintain identical parameter counts. The resulting accuracy or MAE degradation quantified each component's contribution.

Grad-CAM was applied to the last convolutional layers to visualize spectral regions driving the model's decisions. During backpropagation, channel-wise gradients were averaged and used as weights to generate activation maps, which were then normalized and superimposed on the input spectra. These heatmaps highlight energy intervals most influential for each target, revealing physically interpretable relationships between local spectral transitions and macroscopic properties.

S2. Structure Inference Details

S2.1 Oxidation State Determination and Stoichiometric Formula Generation

The first step of the structure candidate screening pipeline is to determine the oxidation states of transition metal elements using their XANES spectra, which serves as the primary chemical constraint for subsequent database retrieval. Each spectrum is processed through the trained SpecFusionNet model to predict oxidation states based on near-edge shifts and spectral feature patterns.

For non-transition metal elements, commonly accepted valence states are assigned based on periodic trends and chemical intuition (e.g., Ca^{2+}, Al^{3+}, O^{2-}), ensuring global charge neutrality within each compound.

After oxidation states are determined, potential stoichiometric formulas satisfying charge neutrality are generated by solving integer charge-balance equations. Each element count is restricted to ≤15 to ensure realistic compositions, and redundant integer multiples are removed.

Taking Ca_2MnAlO_5 material inference as an example, the process is as follows:

1) For the transition metal in this material (the chemical formula of the material is unknown), namely Mn, its XANES spectrum is predicted by machine learning to obtain Mn^{3+}. For non-transition metals, we refer to the periodic table and use chemical intuition to assign generally accepted valence states, obtaining Ca^{2+}, Al^{3+}, O^{2-}.

2) With the oxidation states determined (Ca^{2+}, Mn^{3+}, Al^{3+}, O^{2-}), the next step is to generate stoichiometric formulas that satisfy charge neutrality. The general form of the compound is $Ca_xMn_yAl_zO_w$. The charge balance equation is :

$2x + 3y + 3z - 2w = 0$

This equation ensures that the total positive charge from Ca, Mn and Al balances the total negative charge from oxygen. We look for integer solutions to this equation where each element has no more than 15 atoms. Additionally, we exclude formulas that are simple integer multiples of other formulas to avoid redundancy. Among the possible solutions, we select those that are chemically plausible. For instance, in the case of $CaMnAlO_4$, the subscripts x=1, y=1, z=1, w=4 satisfy the charge balance equation, and similarly Ca_2MnAlO_5 also satisfies the charge balance equation.

S2.2 Crystal System Classification and Template Retrieval

PXRD patterns are analyzed to determine crystal-system labels (cubic, tetragonal, orthorhombic, hexagonal, trigonal, monoclinic, or triclinic) using a CNN-Transformer classifier.

The combination of predicted crystal system and stoichiometric candidates defines the search constraints for structural template retrieval.

Candidate templates are retrieved from the full inorganic crystal structure database of the Materials Project (the framework is also compatible with the Inorganic Crystal Structure Database, ICSD) based on the following hierarchical criteria:

1. **Primary constraint:** Crystal system exactly matching the classification result from the PXRD encoder;
2. **Secondary constraint:** Elemental composition type and valence states consistent with the oxidation states inferred from XANES spectra, satisfying global charge neutrality;
3. **Tertiary constraint:** Coordination geometry of the metal centers comparable to the coordination number predicted from XANES, with priority given to thermodynamically stable structures with negative formation energy.

Template candidates are further ranked by ionic-radius compatibility and coordination similarity to minimize lattice distortion.

S2.3 DFT Optimization and Final Structure Validation

The selected template undergoes **DFT optimization** to refine atomic positions and lattice constants. The optimized structure is validated by comparing simulated and experimental spectra: PXRD (via VASP) and XANES (via FEFF10).

The structure with the highest spectral consistency, quantified by R_PXRD > 0.50, is considered the most probable configuration.

This closed-loop workflow, from oxidation-state prediction and charge-balanced formula generation to structural template retrieval, DFT refinement and spectral validation, ensures a systematic, reproducible, and physically interpretable conditional data-mining pipeline for structure candidate screening and matching of unknown inorganic samples, without requiring prior structural knowledge.

S2.4 Handling Systems with Limited Templates

When structural templates are scarce or absent in databases, the matching constraints are relaxed by permitting element substitutions among chemically analogous species within the same periodic group or with comparable ionic radii. In cases where no valid structural templates are

available in existing databases, the framework can relax the matching constraints by permitting element substitutions among chemically analogous species within the same periodic group or with comparable ionic radii. We explicitly note that de novo structure search methods (e.g., stochastic search, genetic algorithms, or modular assembly) are not implemented in the current version of the framework, and these approaches are only proposed as a future extension to enhance the adaptability of the framework for materials with previously undocumented crystal motifs.

S3. Computational Details

S3.1 Calculation Details of XANES Spectra

In this study, the XANES spectra were calculated using the FEFF10 code[1], a widely used software package for simulating XANES spectra based on the multiple scattering theory. The input file for FEFF10 was carefully constructed to accurately model the electronic structure and scattering processes in the material. The parameters in the input file were set as follows:

The COREHOLE parameter is set to NONE, indicating that corehole effects are not considered in this calculation. The EDGE parameter is set to K, specifying that the calculation is for the K absorption edge. The RSIGMA parameter is included to control the energy resolution of the calculation. The CONTROL parameters are set to 1 1 1 1 1 1, enabling various aspects of the calculation such as different types of scattering processes. The SCF parameters are set to 5.0 0 50 0.1 3, specifying the convergence criteria and maximum number of iterations for the self-consistent field calculation. The FMS parameters are set to 6.0 1, defining the sphere radius and energy mesh for the full multiple scattering calculation. The AFOLP parameter is set to 0.9, controlling the Fermi level broadening. The XANES parameters are set to 4 0.05 0.1, specifying the number of points, energy step, and broadening for the XANES calculation. These parameters were chosen based on a combination of theoretical considerations and empirical optimization to ensure reliable and meaningful XANES spectra simulations.

S3.2 DFT Structural Optimization

The structural optimization was performed using the Vienna Ab Initio Simulation Package (VASP)[2], a widely used software package for quantum mechanical calculations based on DFT. The parameters for the structural optimization were carefully chosen to balance computational efficiency and accuracy. The EDIFF parameter was set to 1E-5, defining the convergence criterion for the electronic self-consistent loop. The EDIFFG parameter was set to -0.02, and the ENCUT was set to 400. The IBRION parameter was set to 2, selecting the conjugate gradient algorithm for the ionic relaxation. The ISIF parameter was set to 3, allowing for full optimization of both the ion positions and the cell shape and volume. These parameters were chosen based on a combination of theoretical considerations and empirical optimization to ensure reliable and meaningful structural optimization results.

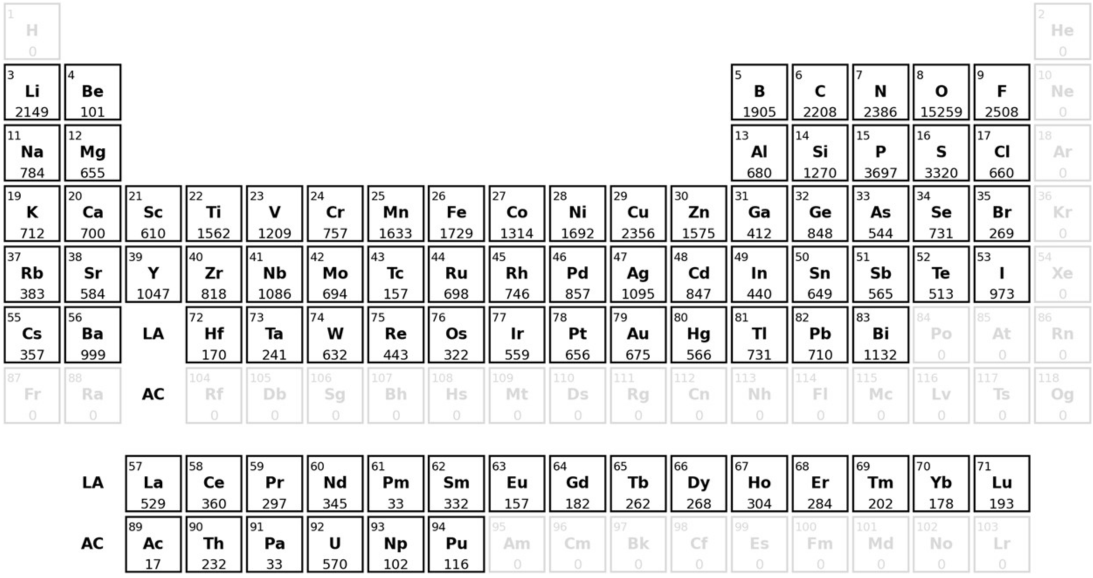

Figure S1. Periodic table showing elements included in the XANES dataset. The top-left number in each cell represents the atomic number, and the bottom number indicates occurrence frequency. Black cells denote elements present in the dataset, while gray cells denote absent ones.

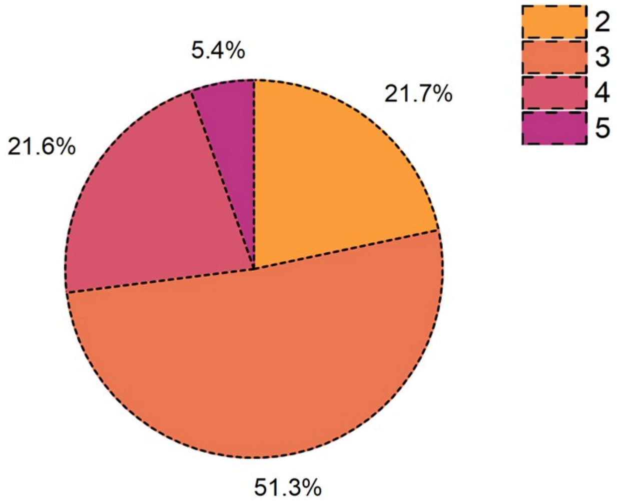

Figure S2. Distribution of compounds containing different numbers of elements in the dataset.

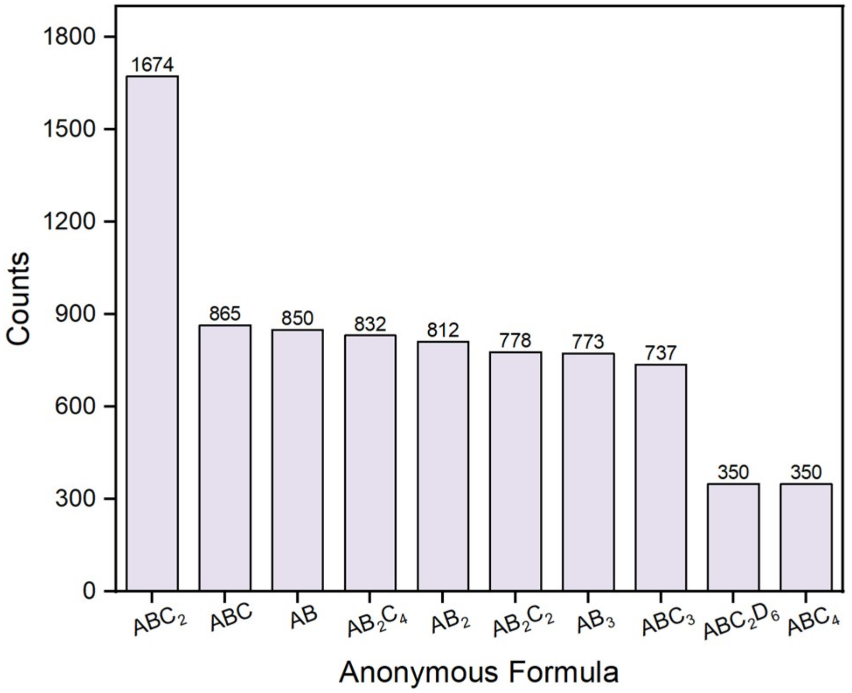

Figure S3. Frequency distribution for the top ten anonymous chemical formulas in the dataset.

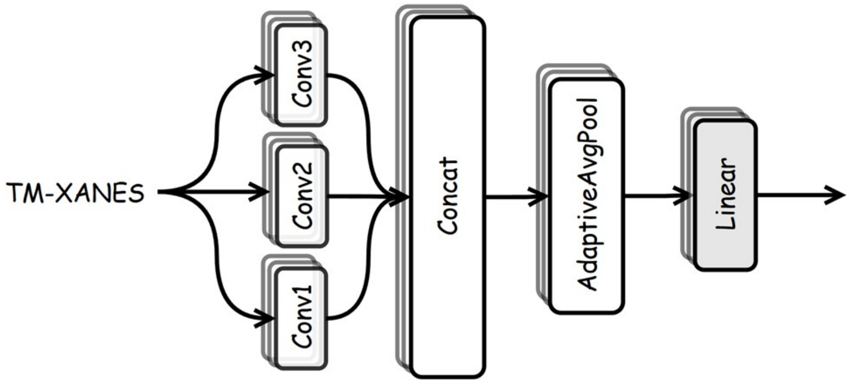

Figure S4. Schematic diagram of the multi-scale feature extractor in SpecFusionNet, consisting of three convolutional branches (Conv1-Conv3) followed by feature concatenation and a linear output layer.

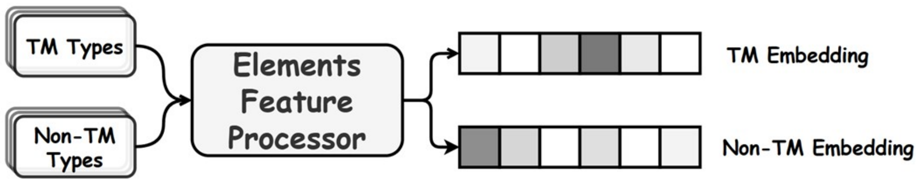

Figure S5. Schematic of the Element Feature Processor within SpecFusionNet. TM and non-TM elements are processed through separate embedding branches before fusion with spectral features for property prediction.

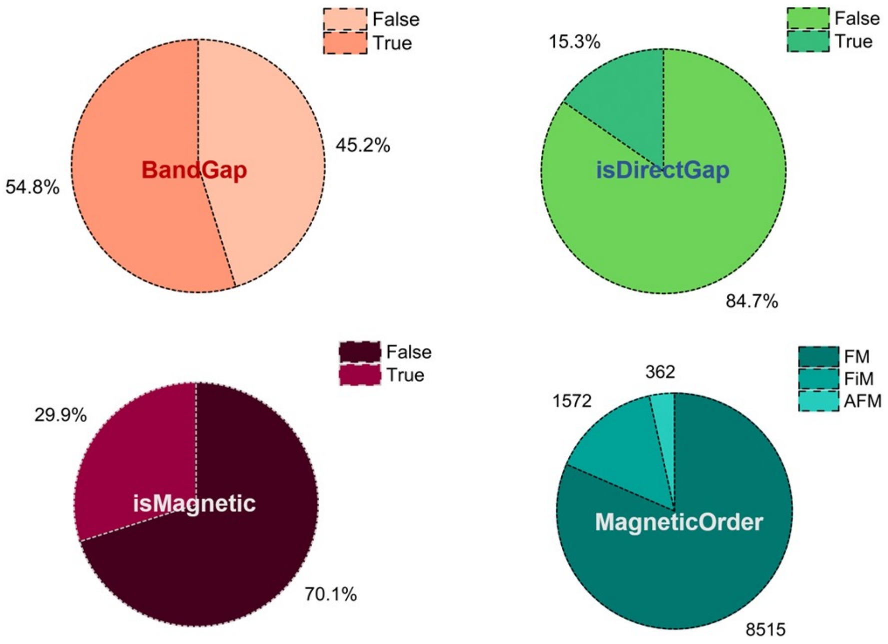

Figure S6. Statistical distribution of the four classification tasks in the dataset.

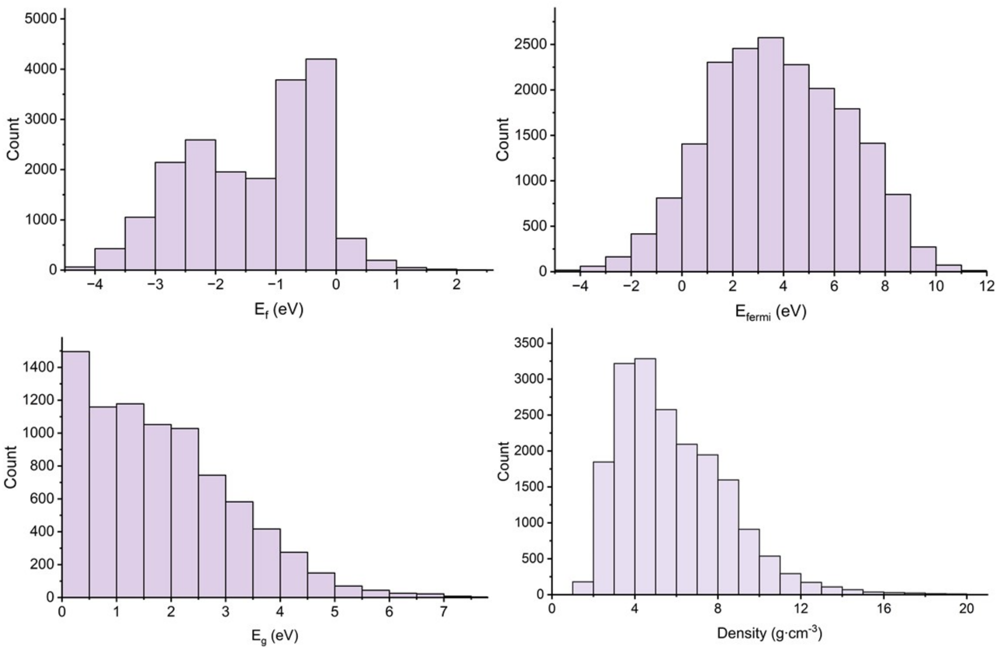

Figure S7. Distributions of regression tasks (E_f, E_{fermi}, E_g, Density) within transition-metal materials.

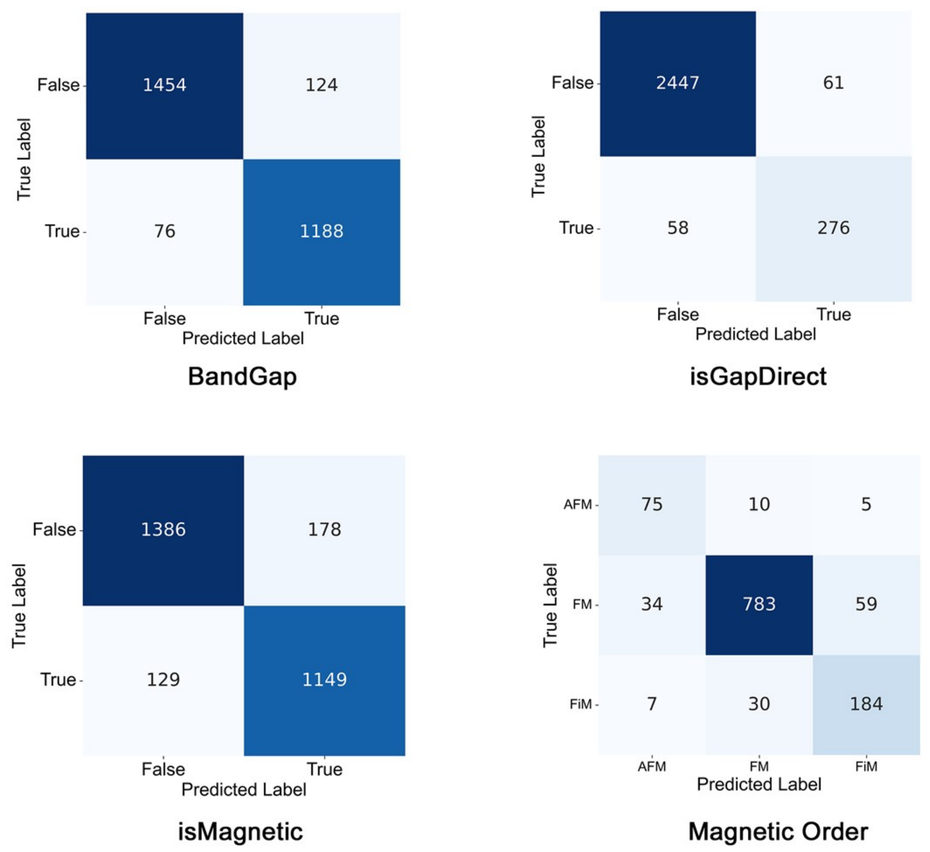

Figure S8. Confusion matrices of classification tasks (BandGap, isGapDirect, isMagnetic, Magnetic Order) within transition-metal materials.

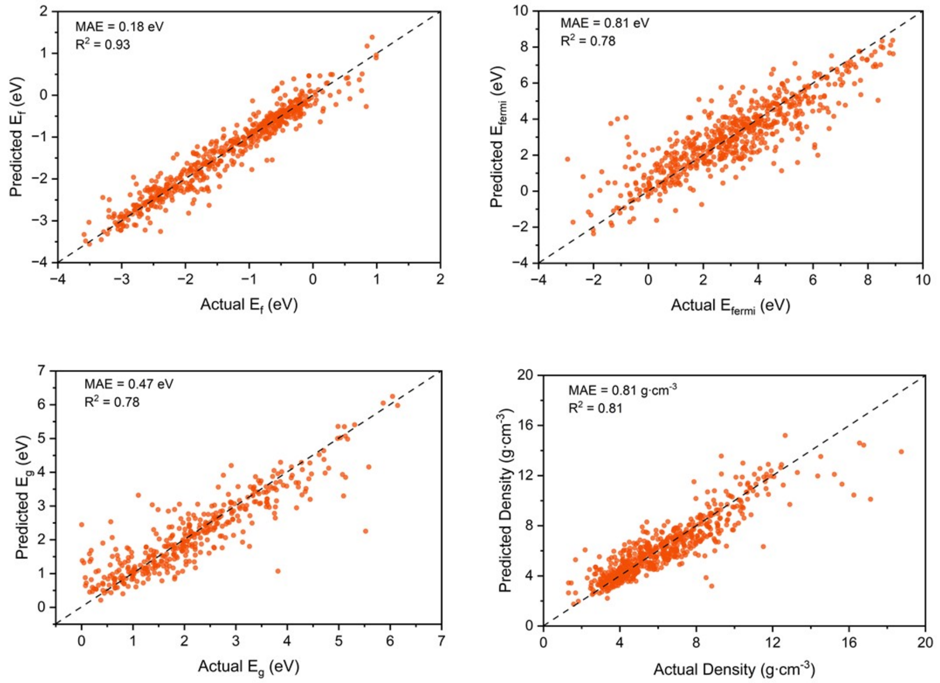

Figure S9. Predicted vs. actual values for four regression tasks within alkaline-earth metal materials.

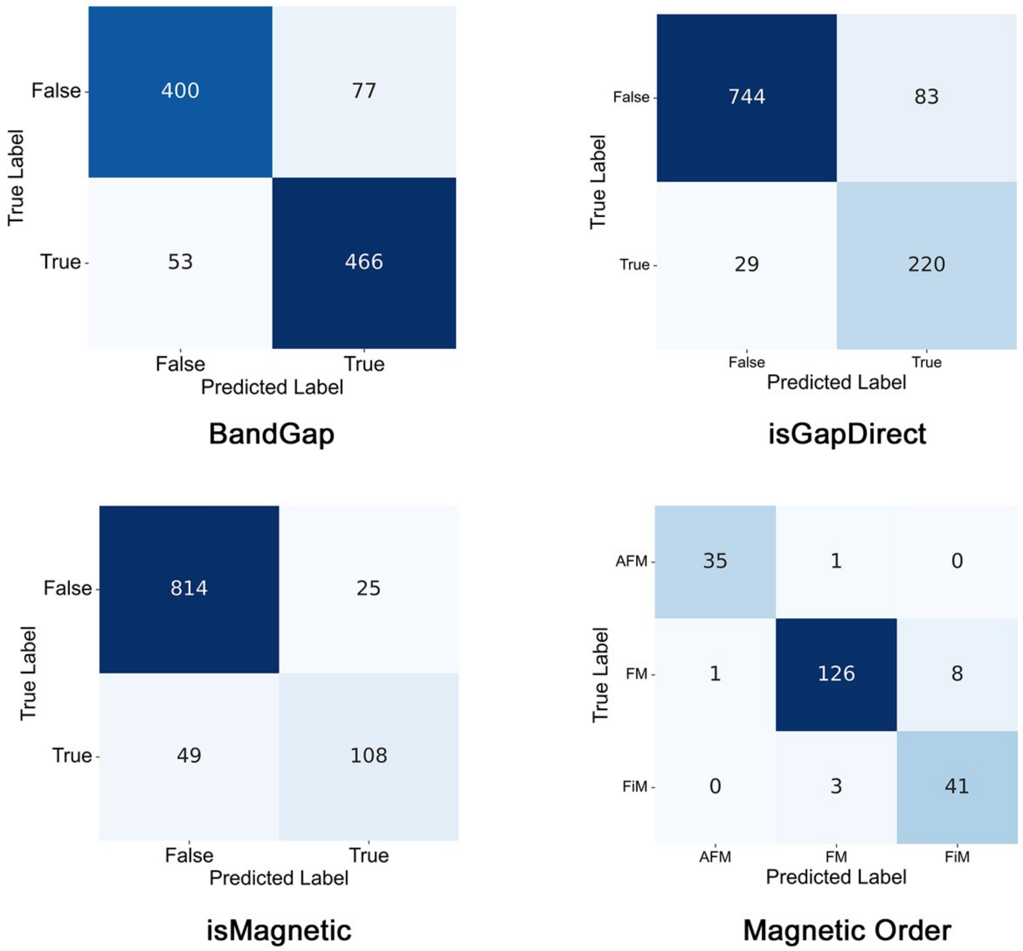

Figure S10. Confusion matrices of classification tasks within alkaline-earth metal materials.

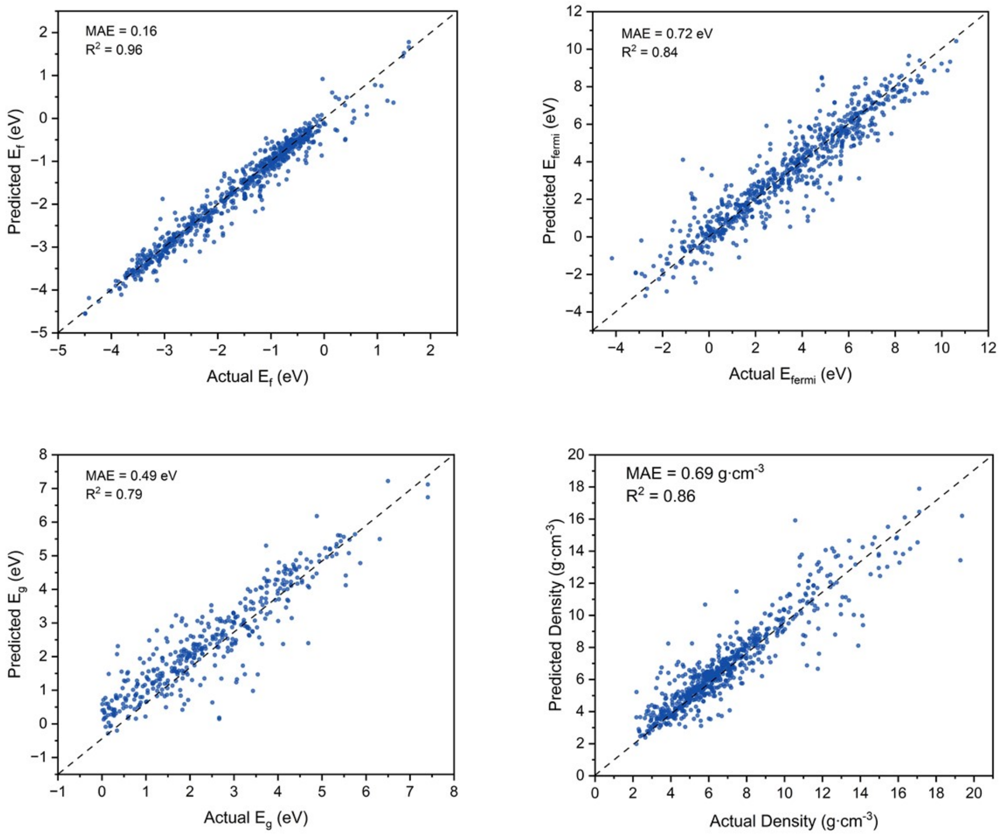

Figure S11. The predicted and actual values of four regression tasks using $L_{2,3}$-edge XANES spectra of 5d transition metals and lanthanides.

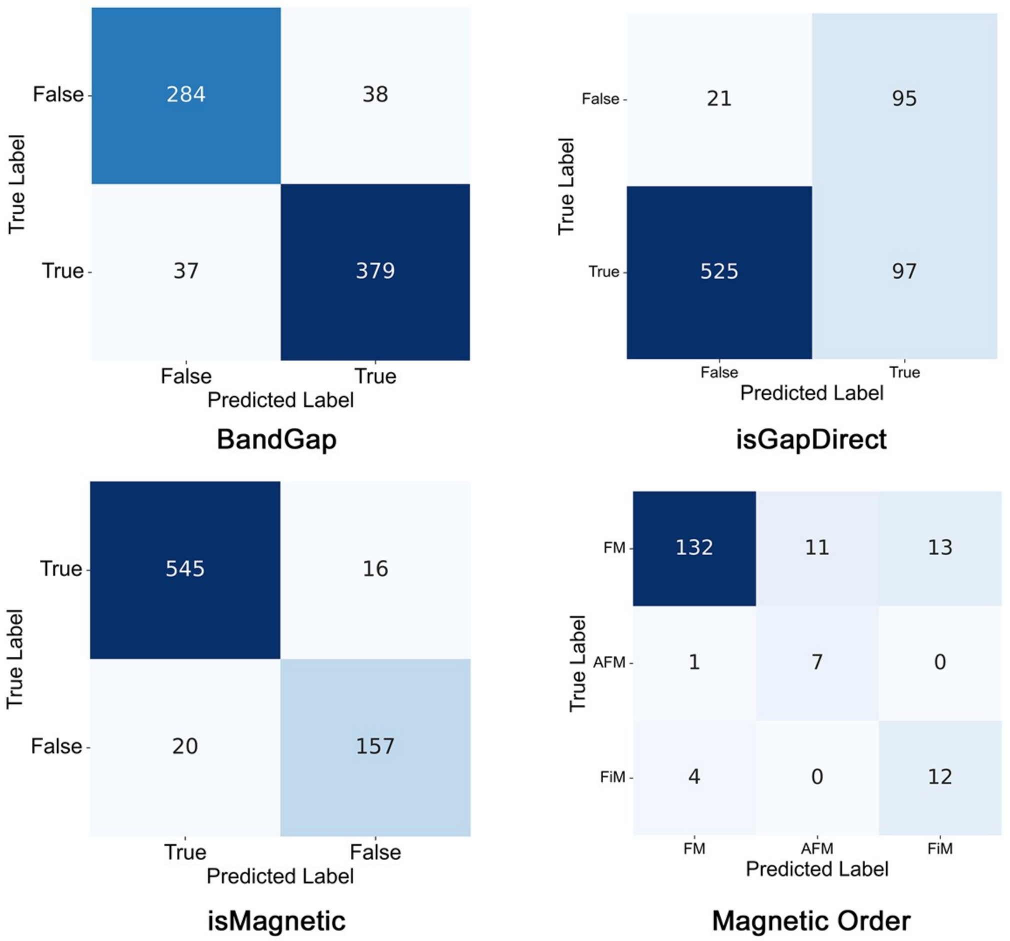

Figure S12. Confusion matrices of classification tasks for 5d transition metals and lanthanides.

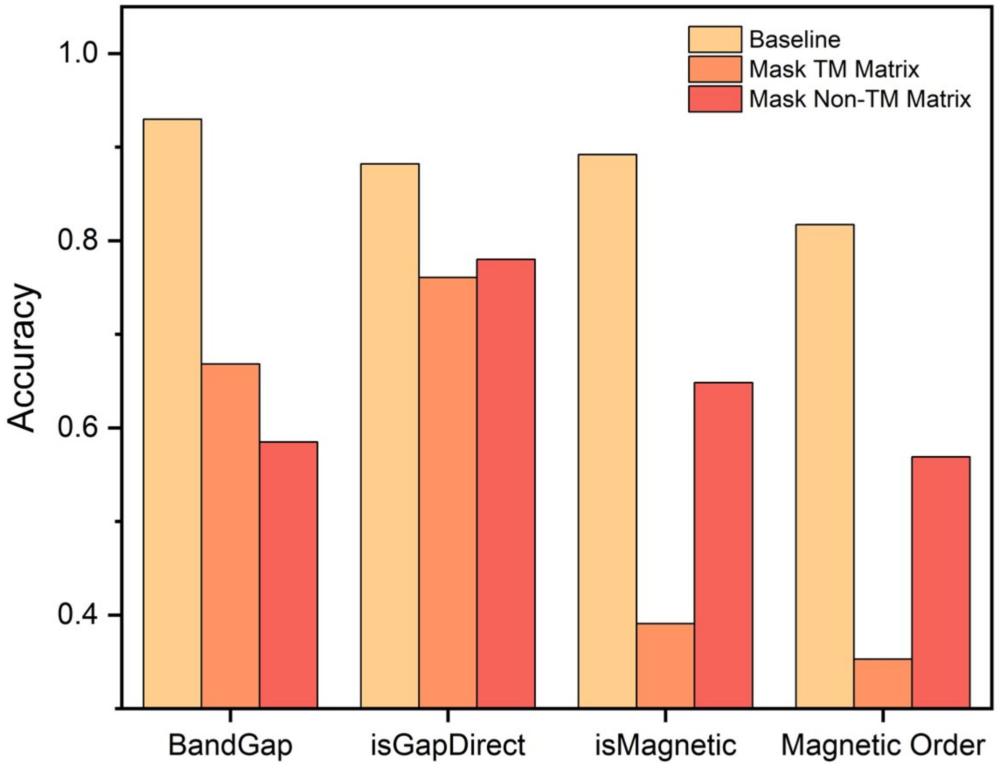

Figure S13. Ablation study comparing different element-fusion matrices across four classification tasks. The bars show model accuracy for the baseline, masked transition-metal fusion matrix, and masked non-transition-metal fusion matrix.

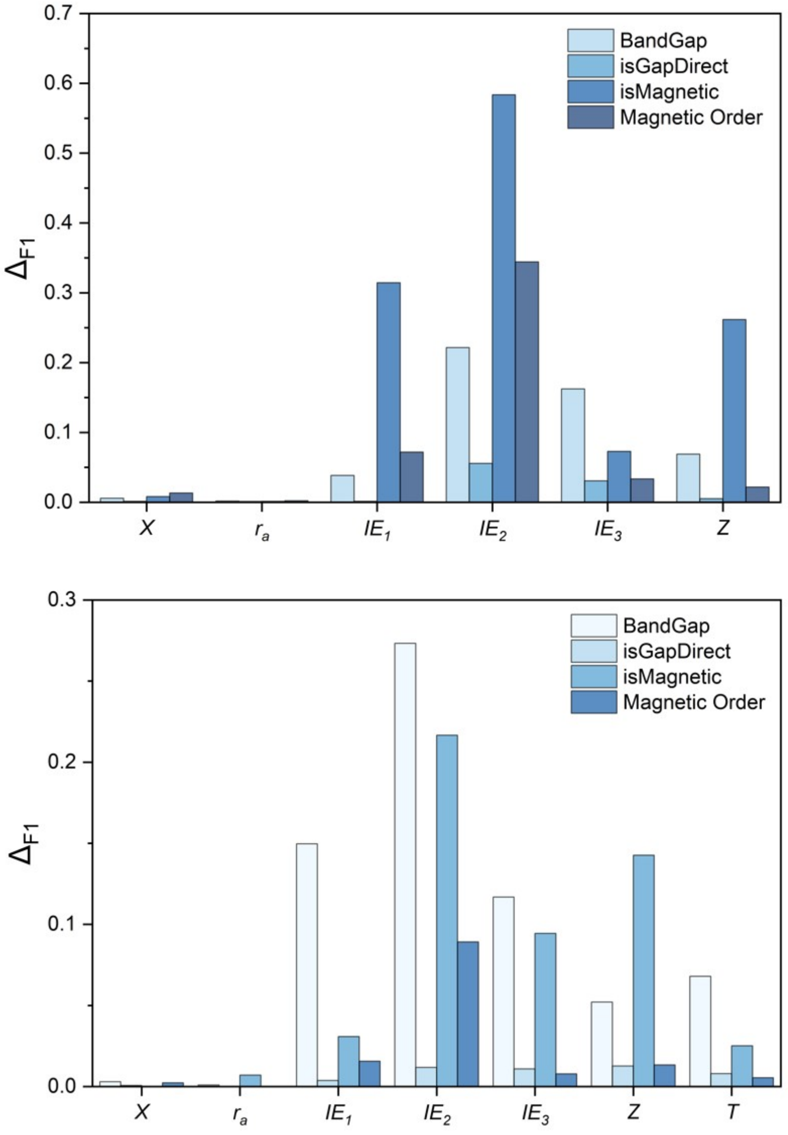

Figure S14. Ablation results of embedded atomic descriptors in transition metals (top) and non-transition metals (bottom). Bars indicate the change in F1 score after descriptor removal.

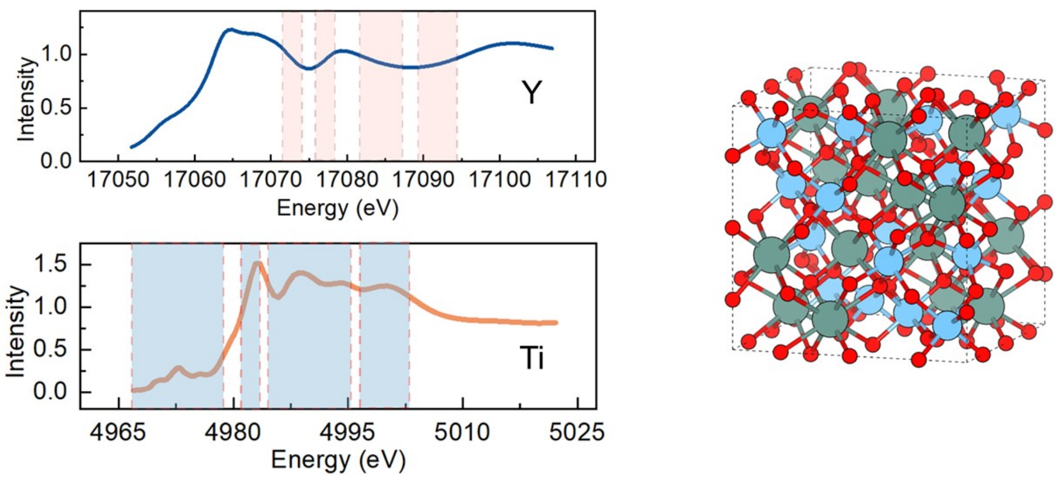

Figure S15. Grad-CAM visualization of Y and Ti K-edge XANES spectra of $Y_2Ti_2O_7$ (mp-5373), highlighting the spectral regions contributing to the density prediction. The shaded areas in the spectra indicate the most influential energy regions identified by the model. The right panel shows the corresponding atomic structure of $Y_2Ti_2O_7$.

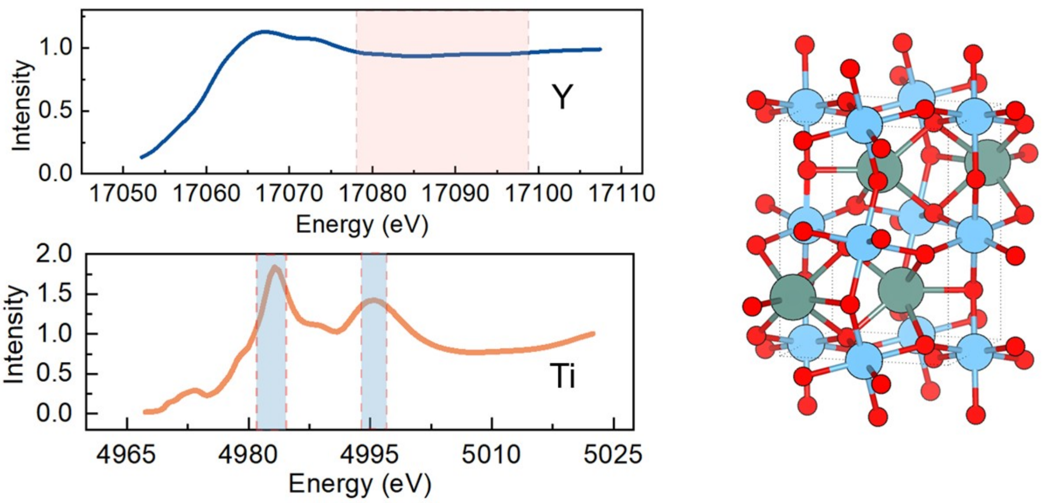

Figure S16. Grad-CAM visualization of Y and Ti K-edge XANES spectra of $YTiO_3$ (mp-4355), highlighting the spectral regions contributing to the density prediction. The shaded areas in the spectra indicate the most influential energy regions identified by the model. The right panel shows the corresponding atomic structure of $YTiO_3$.

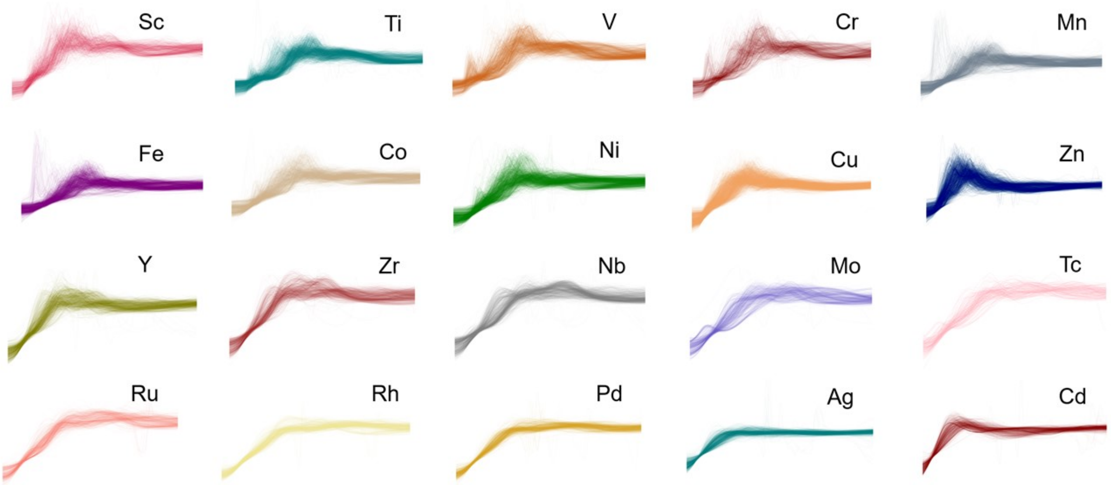

Figure S17. Standardized XANES spectra for all transition metal elements included in the dataset. Each color represents a different element.

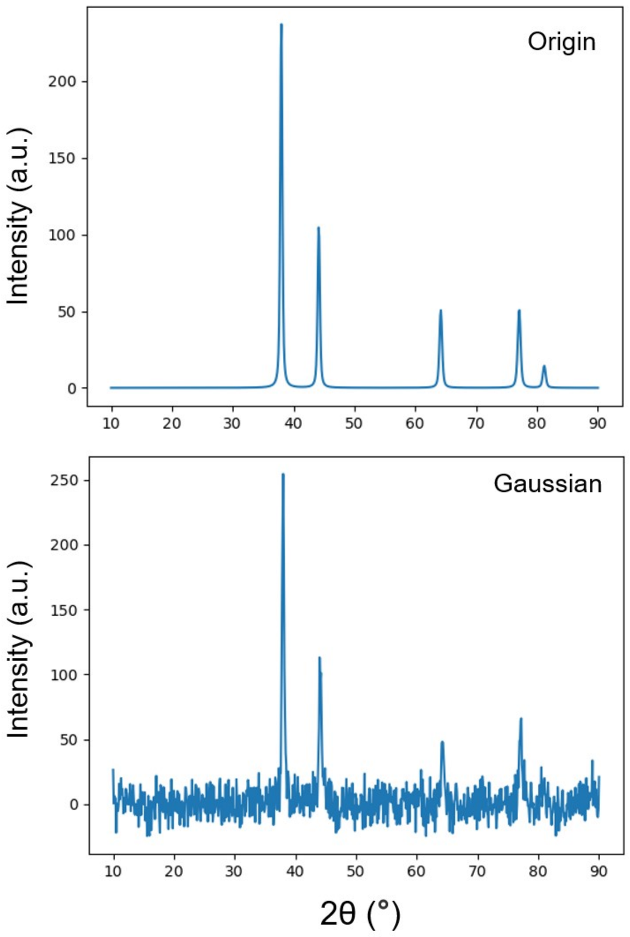

Figure S18. Example of PXRD preprocessing: (top) original simulated PXRD spectrum and (bottom) spectrum after Gaussian noise addition.

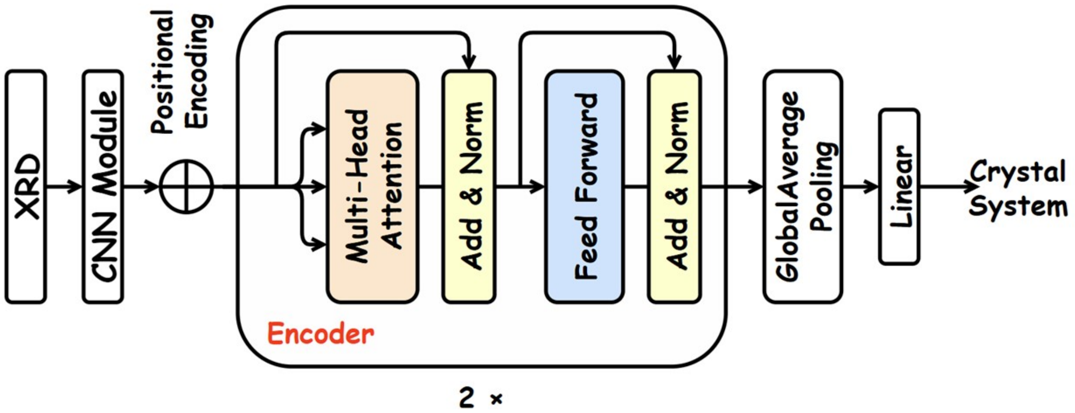

Figure S19. Architecture of the hybrid CNN-Transformer model used for PXRD feature extraction and crystal-system classification.

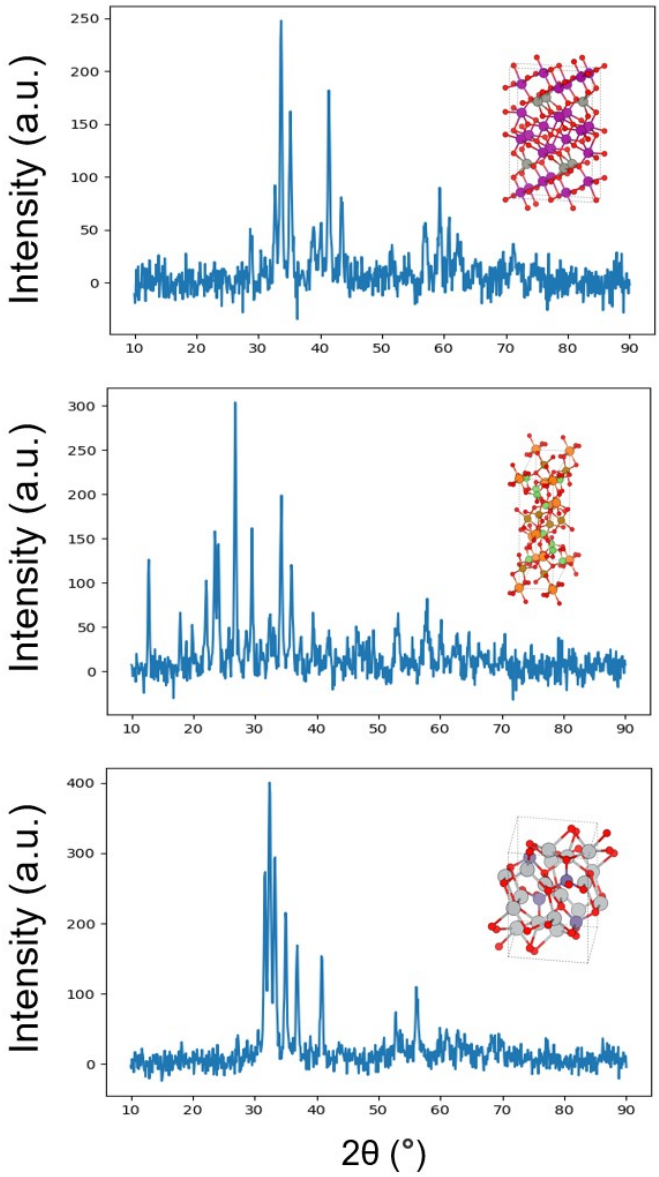

Figure S20. PXRD patterns of Mn_4ZnO_6 (top), $Mg_3Fe_3As_4O_{16}$ (middle), and Ag_4GeO_4 (bottom). The crystal system of Mn_4ZnO_6 is orthorhombic, $Mg_3Fe_3As_4O_{16}$ is monoclinic, and Ag_4GeO_4 is triclinic.

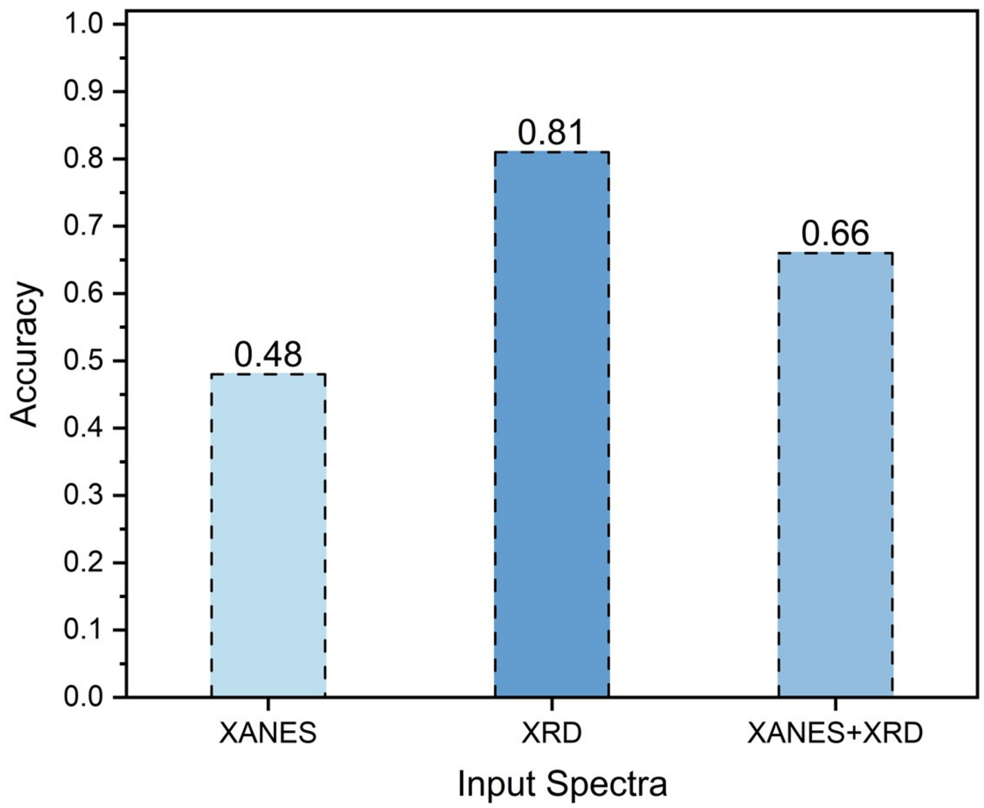

Figure S21. Accuracy of crystal-system prediction using different spectral input combinations (XANES-only, PXRD-only, and XANES+PXRD).

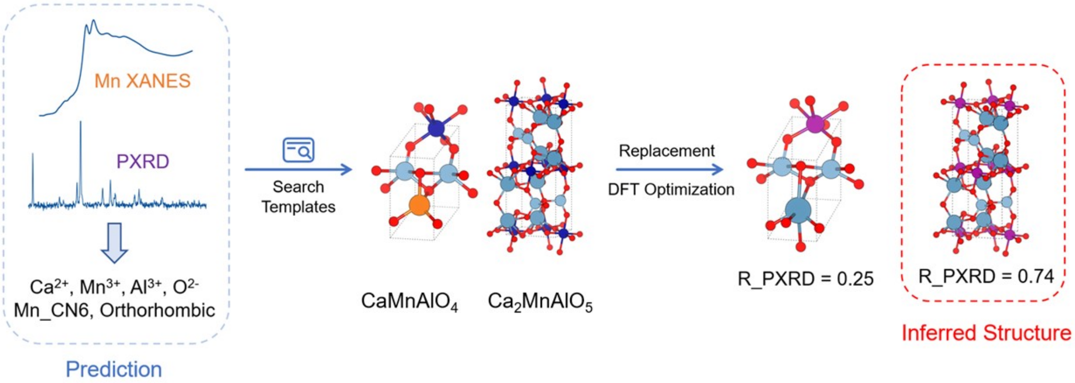

Figure S22. Example of the structure-inference process for Ca_2MnAlO_5, showing the stepwise workflow from spectra to final structure reconstruction.

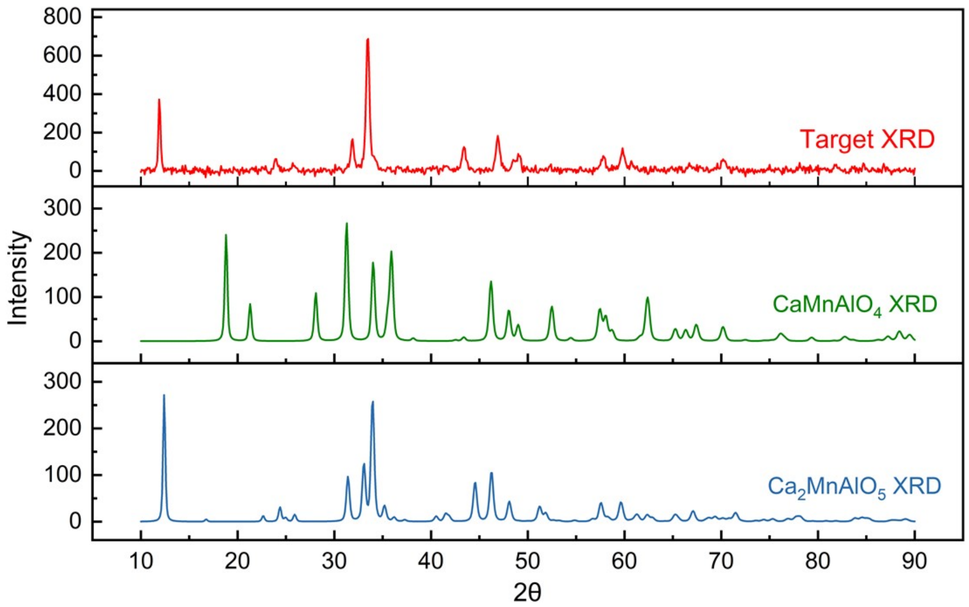

Figure S23. Comparison of PXRD patterns for the target Ca-Mn-Al-O material (red) and two candidate formulas, $CaMnAlO_4$ (green) and Ca_2MnAlO_5(blue).

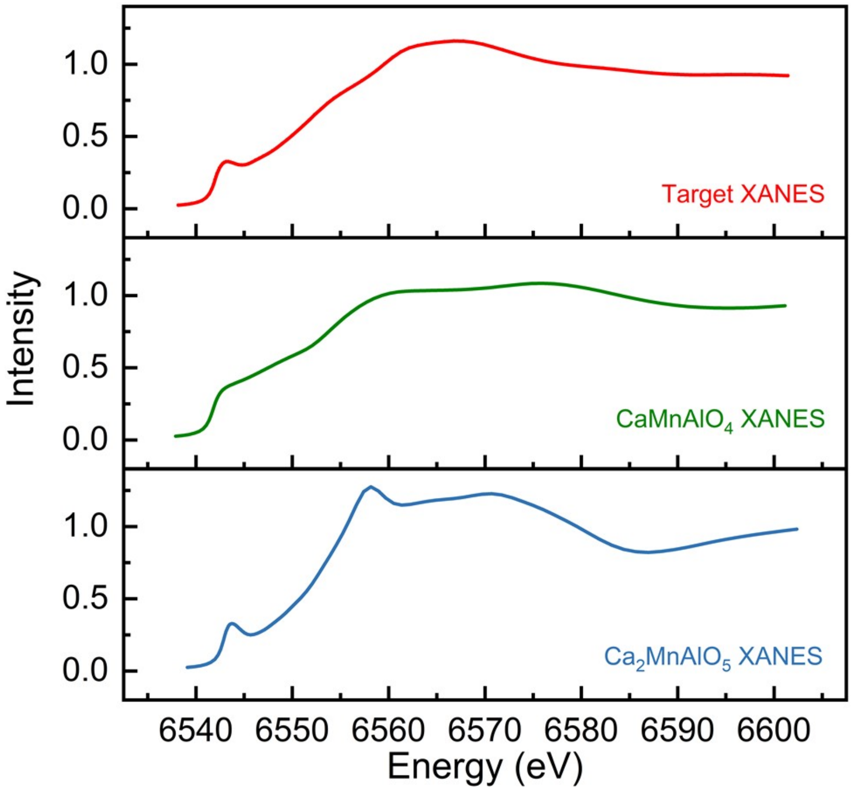

Figure S24. Mn K-edge XANES spectra of the same three materials, target (red), $CaMnAlO_4$ (green), and Ca_2MnAlO_5 (blue), used for spectral matching and validation.

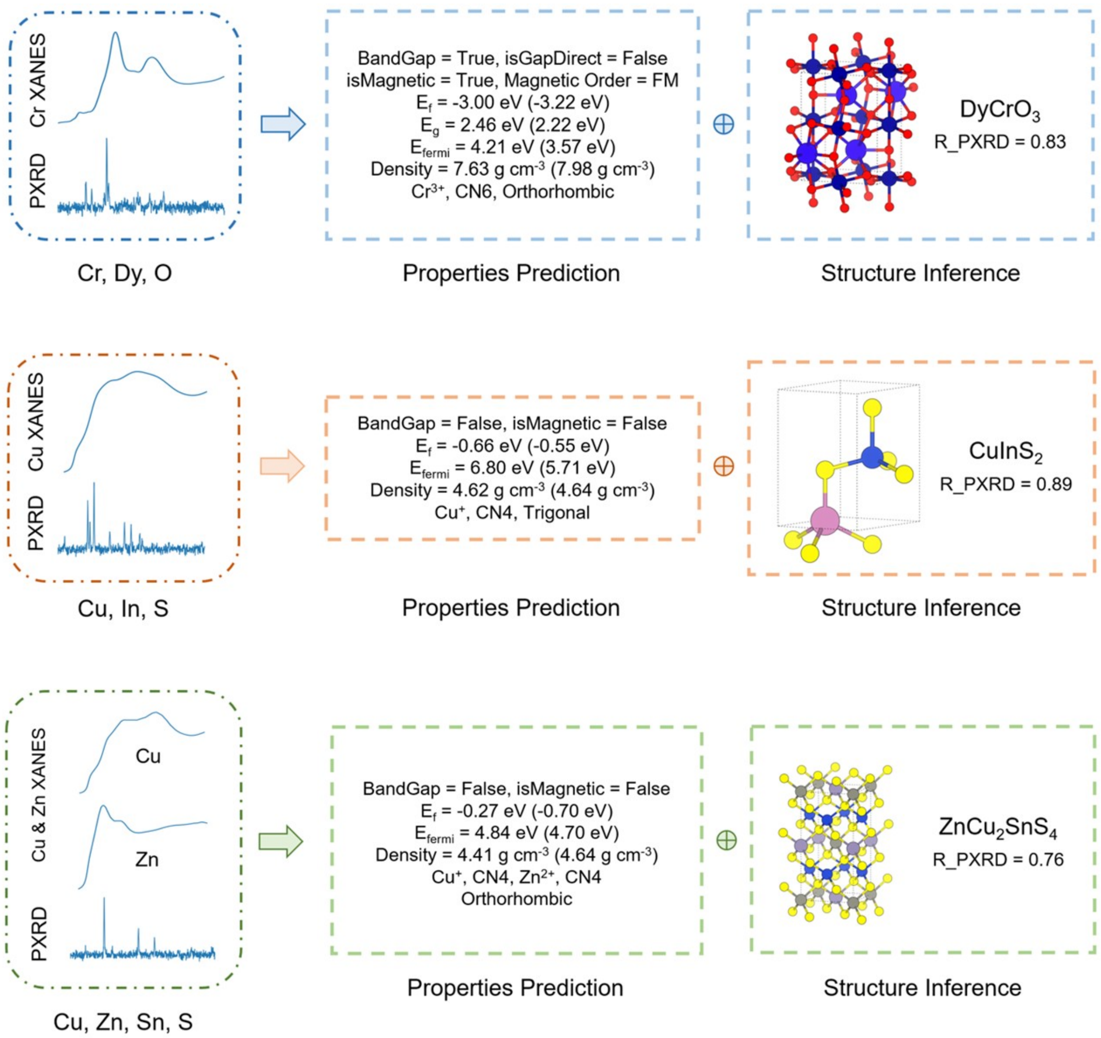

Figure S25. Multi-target property prediction and structure inference for $DyCrO_3$, $CuInS_2$, and $ZnCu_2SnS_4$. Left: input PXRD and XANES spectra. Middle: predicted material properties compared with reference values. Right: inferred crystal structure with the corresponding R_PXRD match parameter.

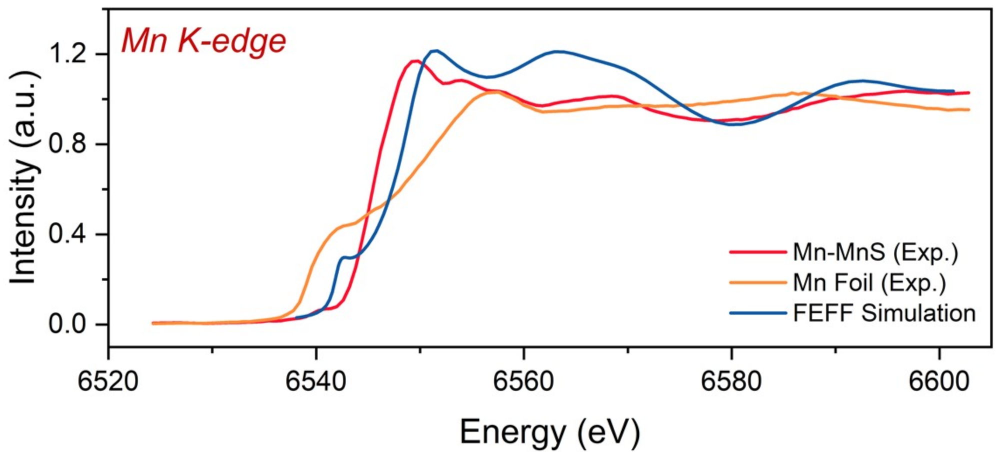

Figure S26. Experimental and simulated Mn K-edge XANES spectra of MnS.

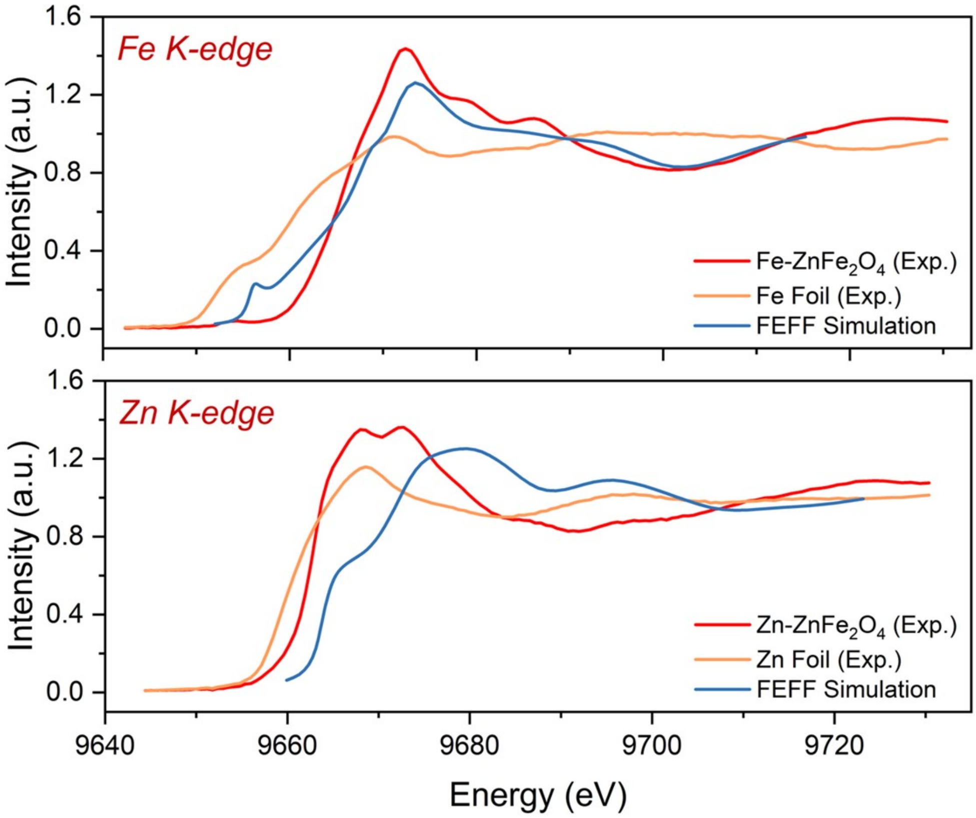

Figure S27. Experimental and simulated XANES spectra of $ZnFe_2O_4$.

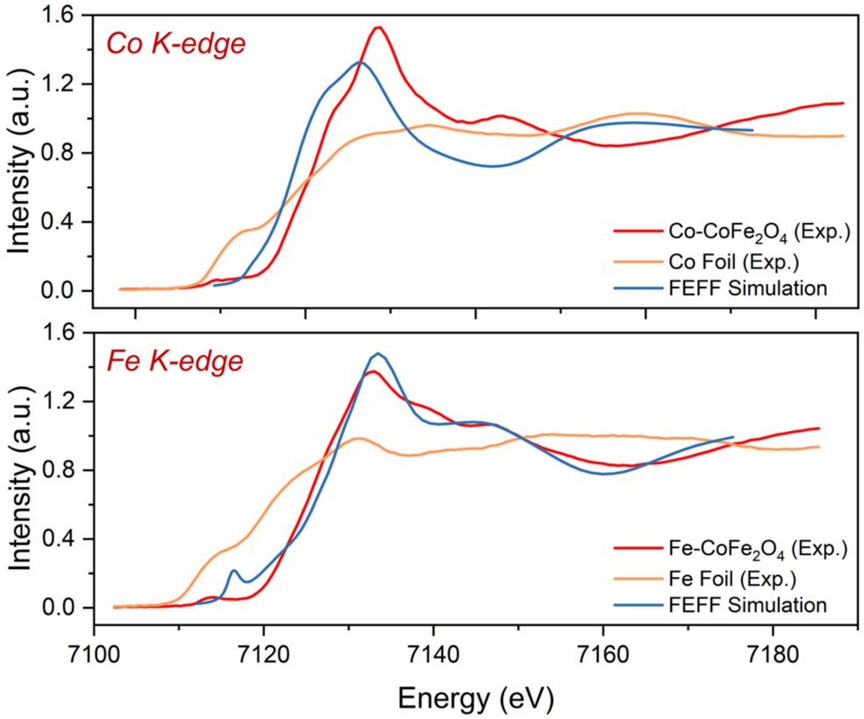

Figure S28. Experimental and simulated XANES spectra of $CoFe_2O_4$.

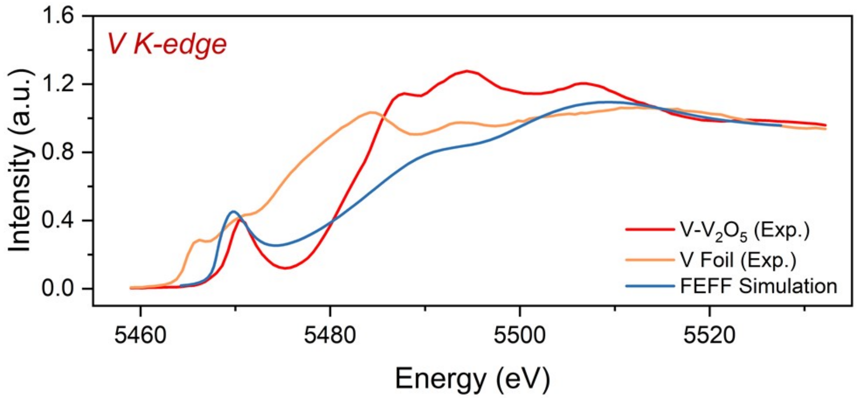

Figure S29. Experimental and simulated XANES spectra of V_2O_5.

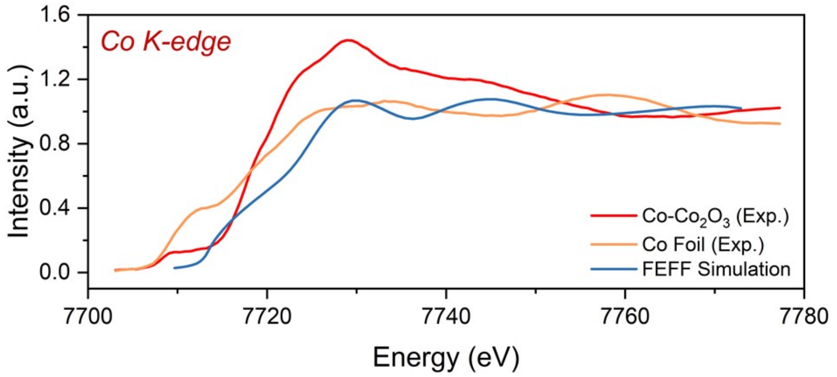

Figure S30. Experimental and simulated XANES spectra of Co_2O_3.

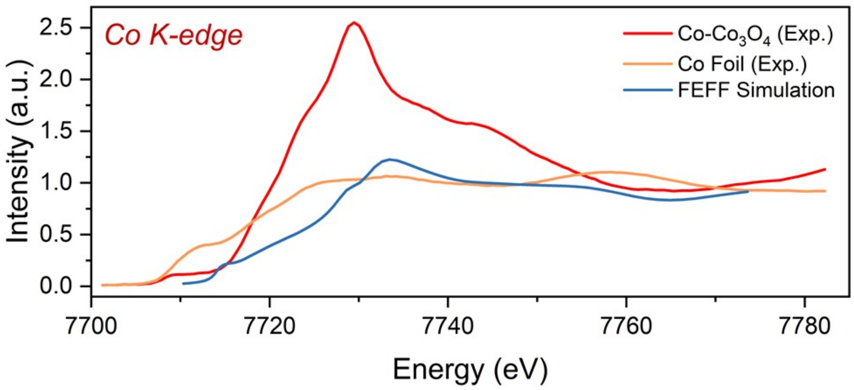

Figure S31. Experimental and simulated XANES spectra of Co_3O_4.

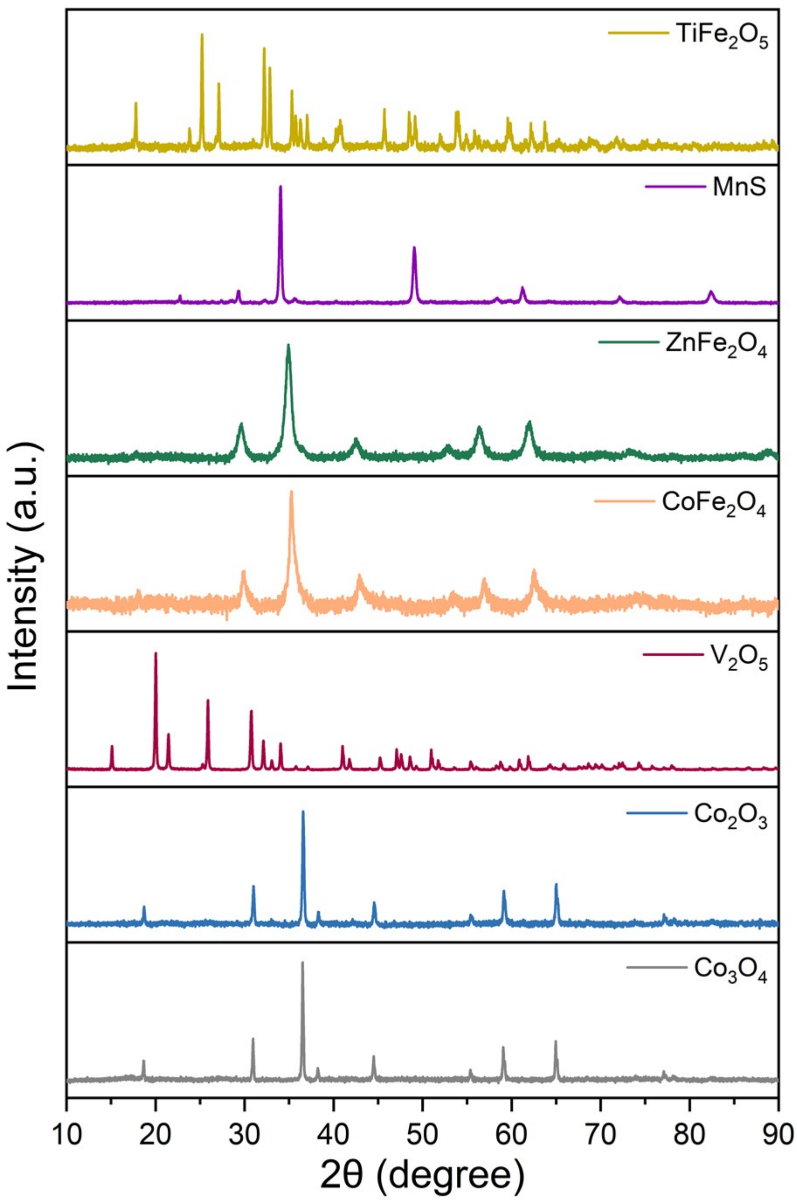

Figure S32. Experimental PXRD patterns of representative materials used for spectral validation.

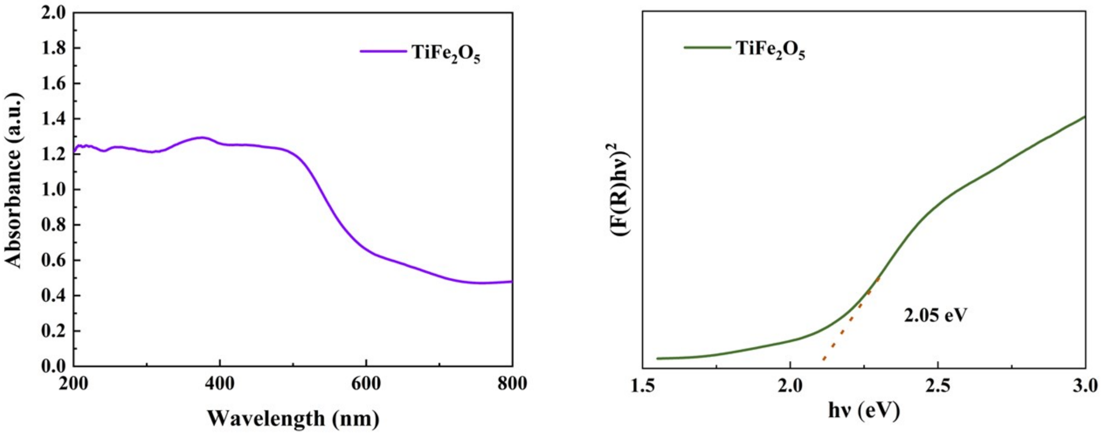

Figure S33. UV-visible absorption spectrum and Tauc-plot band-gap fitting of $TiFe_2O_5$.

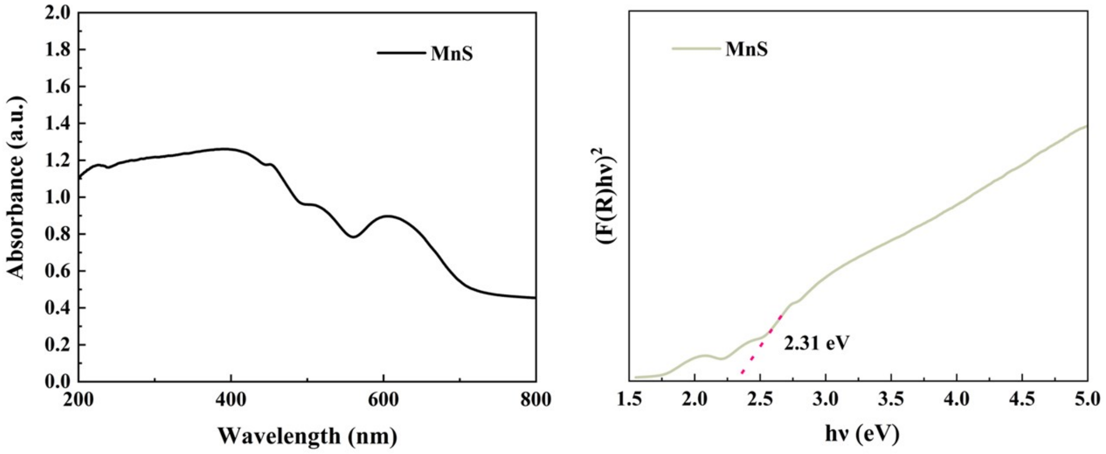

Figure S34. UV-visible absorption spectrum and Tauc-plot band-gap fitting of MnS.

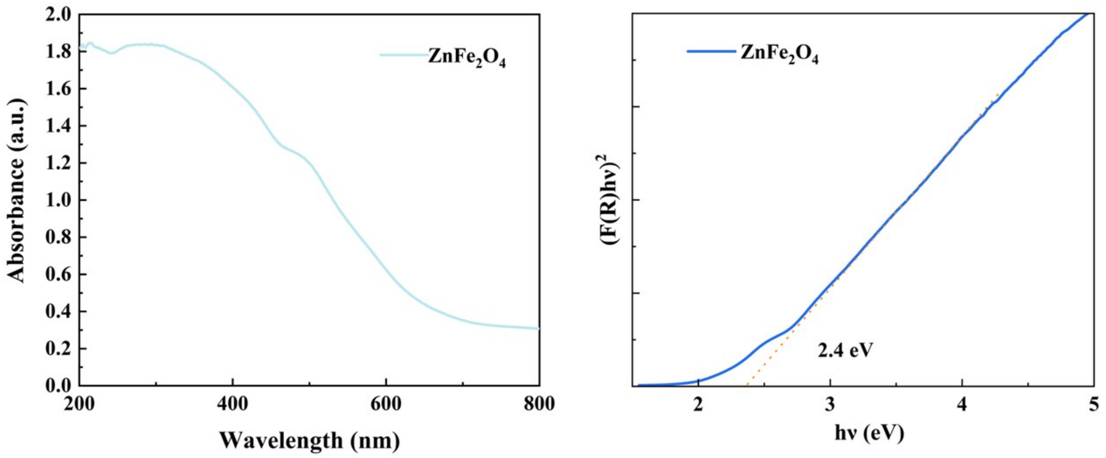

Figure S35. UV-visible absorption spectrum and Tauc-plot band-gap fitting of $ZnFe_2O_4$.

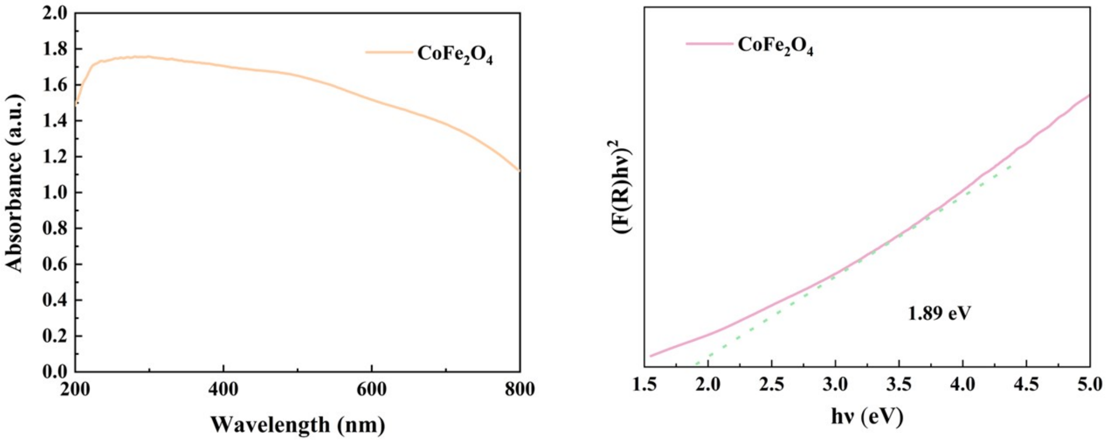

Figure S36. UV-visible absorption spectrum and Tauc-plot band-gap fitting of $CoFe_2O_4$.

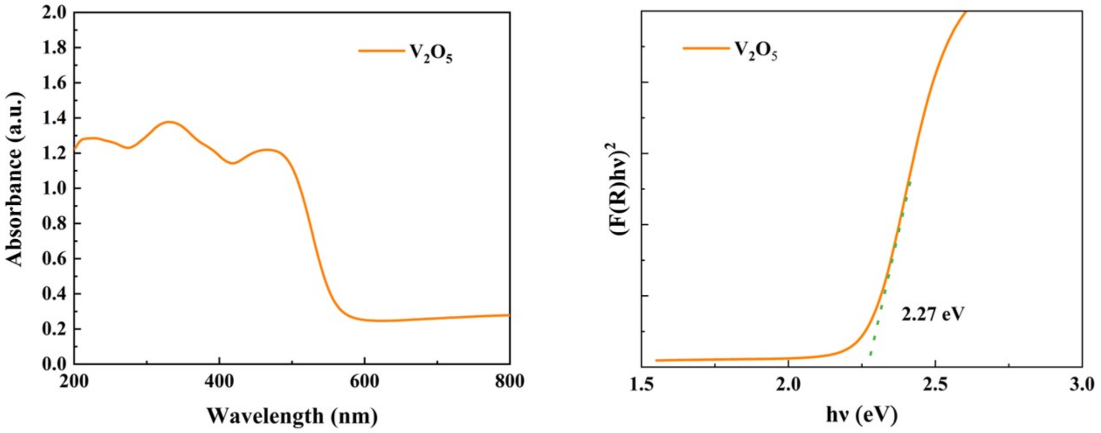

Figure S37. UV-visible absorption spectrum and Tauc-plot band-gap fitting of V_2O_5.

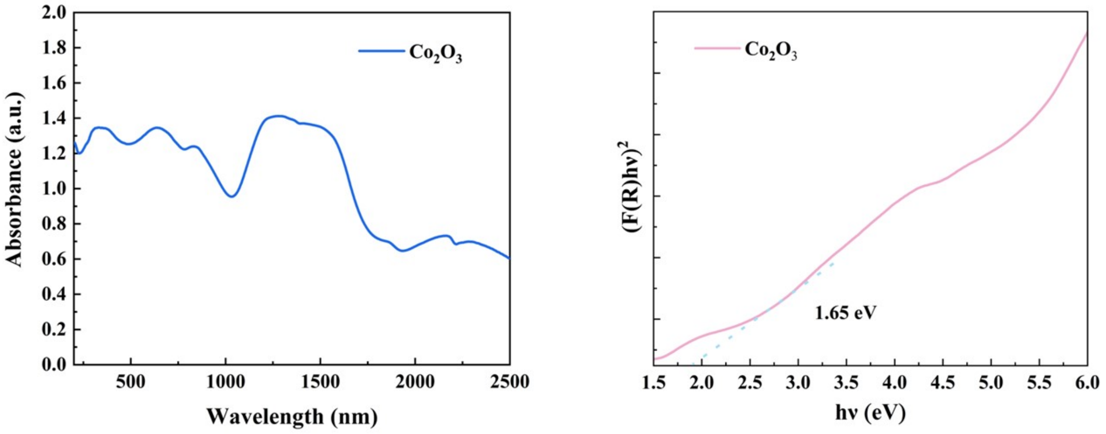

Figure S38. UV-visible absorption spectrum and Tauc-plot band-gap fitting of Co_2O_3.

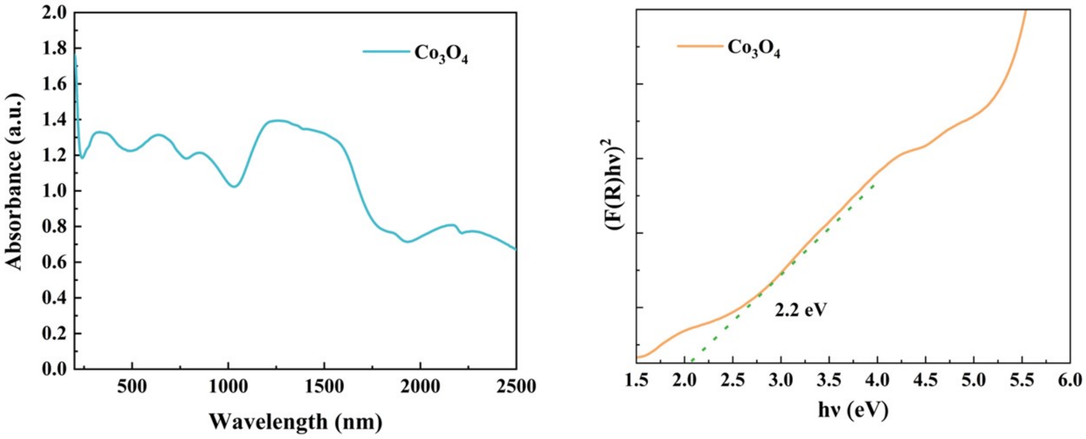

Figure S39. UV-visible absorption spectrum and Tauc-plot band-gap fitting of Co_3O_4.

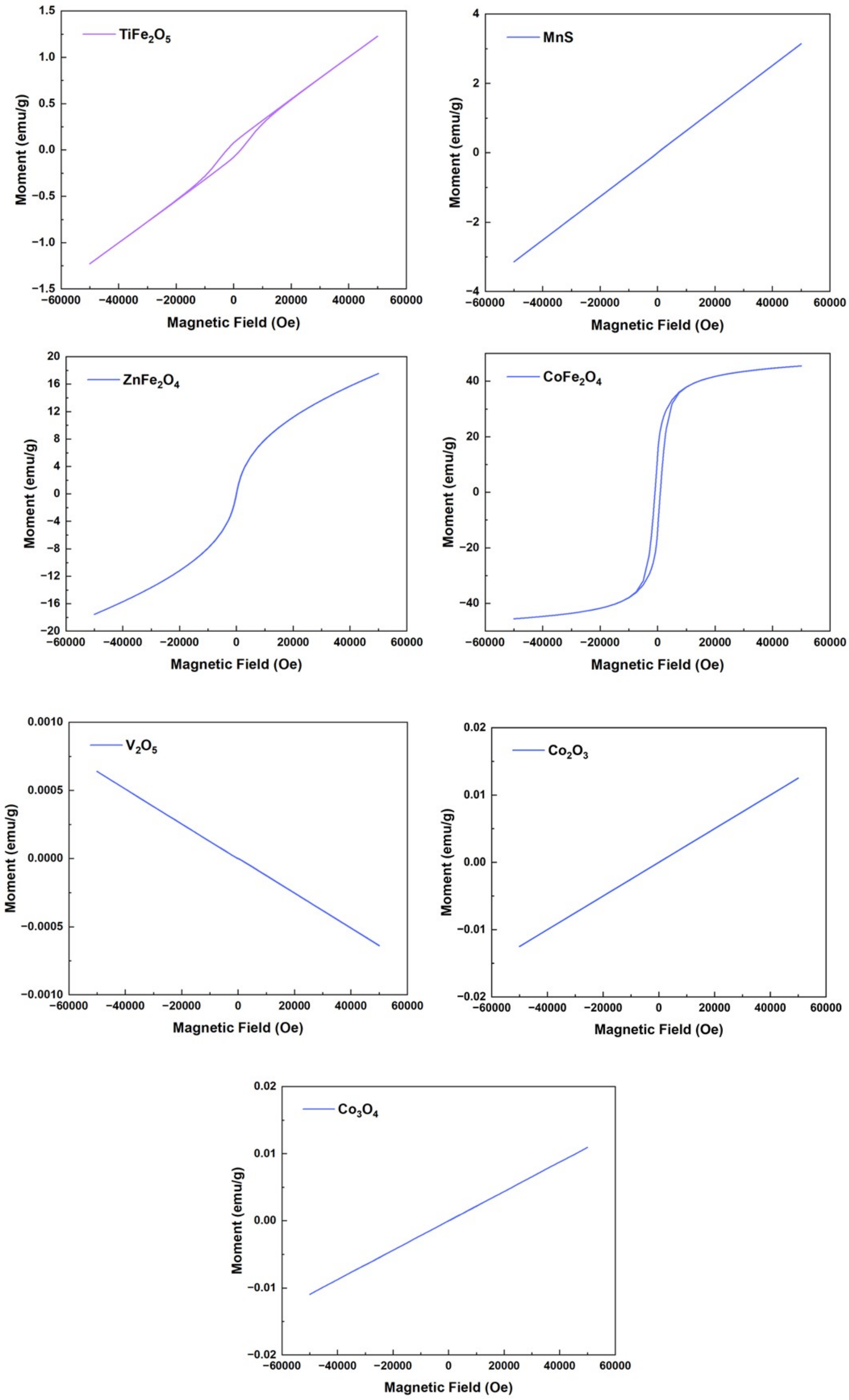

Figure S40. Magnetic hysteresis loops of the seven materials.

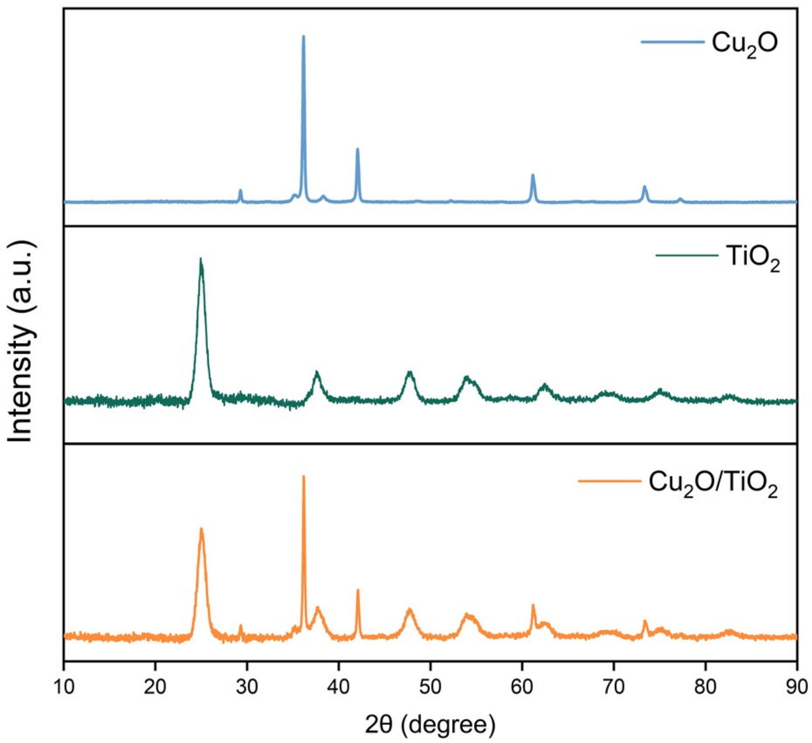

Figure S41. Experimental PXRD patterns of representative materials used for spectral validation.

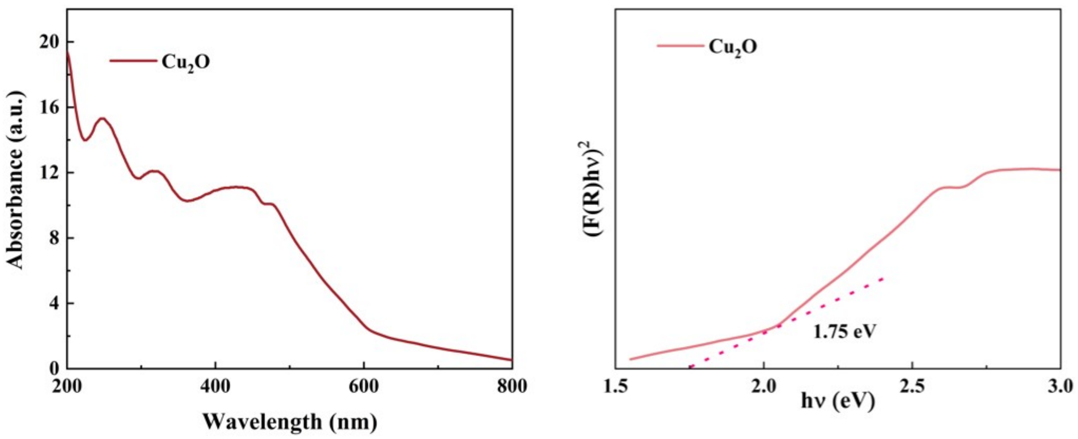

Figure S42. UV-visible absorption spectrum and Tauc-plot band-gap fitting of Cu_2O.

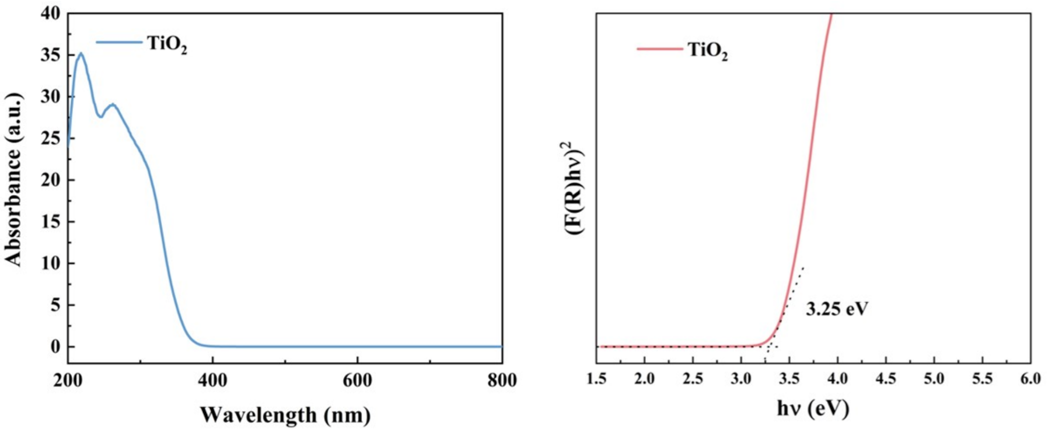

Figure S43. UV-visible absorption spectrum and Tauc-plot band-gap fitting of TiO_2.

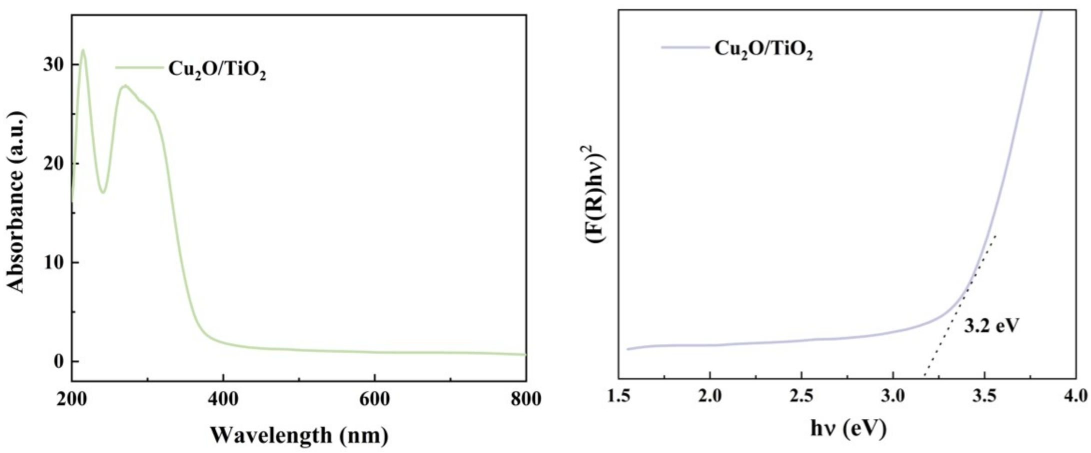

Figure S44. UV-visible absorption spectrum and Tauc-plot band-gap fitting of Cu_2O/TiO_2.

Table S1a. Predicted and Experimental Properties of Seven Materials

**Property**	**$TiFe_2O_5$ (Pred/Exp)**	**MnS (Pred/Exp)**	**$ZnFe_2O_4$ (Pred/Exp)**	**$CoFe_2O_4$ (Pred/Exp)**
Is Band Gap	Yes / Yes	Yes / Yes	Yes / Yes	Yes / Yes
Is Magnetic	Yes / Yes	Yes / No	Yes / Yes	Yes / Yes
Band Gap (eV)	1.33 / 2.05	1.04 / 2.31	1.76 / 2.40	1.39 / 1.89
E_f (eV)	-2.26 / -2.34	-1.16 / -1.13	-1.65 / -1.75	-1.59 / -1.57
Fermi Level (eV)	3.76 / 2.70	4.45 / 3.15	1.65 / 2.30	1.65 / 2.91
Density (g/cm^3)	4.73 / 4.20	3.53 / 3.37	4.37 / 5.34	3.80 / 5.17
CNs	(6, 6) / (6, 6)	4 / 4	(6, 6) / (4, 6)	(4, 6) / (4, 6)
States	(+3, +4) / (+3, +4)	+2 / +2	(+2, +3) / (+2, +3)	(+2, +3) / (+2, +3)
Crystal System	Orth. / Orth.	Cubic / Cubic	Cubic / Cubic	Cubic / Cubic

Table S1b. Predicted and Experimental Properties of Seven Materials

**Property**	**V_2O_5 (Pred/Exp)**	**Co_2O_3 (Pred/Exp)**	**Co_3O_4 (Pred/Exp)**
Is Band Gap	Yes / Yes	Yes / Yes	Yes / Yes
Is Magnetic	No / No	Yes / No	No / No
Band Gap (eV)	1.75 / 2.27	1.26 / 1.65	1.78 / 2.20
E_f (eV)	-2.06 / -2.29	-1.14 / -0.93	-1.04 / -1.28
Fermi Level (eV)	-1.60 / -0.54	3.00 / 1.96	2.44 / 3.16
Density (g/cm^3)	2.99 / 3.44	3.40 / 4.87	4.31 / 5.52
CNs	5 / 5	6 / 6	6 / (4, 6)
States	+5 / +5	+3 / +3	+3 / (+3, +2)
Crystal System	Orth. / Orth.	Cubic / Cubic	Cubic / Cubic

REFERENCES

1. J. J. Kas, F. D. Vila, C. D. Pemmaraju, T. S. Tan and J. J. Rehr, *J Synchrotron Radiat*, 2021, **28**, 1801-1810.
2. G. Kresse and J. Furthmüller, *Physical Review B*, 1996, **54**, 11169-11186.
